# Supplementary figures and images for: A novel lineage-tracing mouse model for studying early MmuPV1 infections
Source: eLife. 2022 May 9;11:e72638. doi: 10.7554/eLife.72638 (PMC9084889; doi:10.7554/eLife.72638)

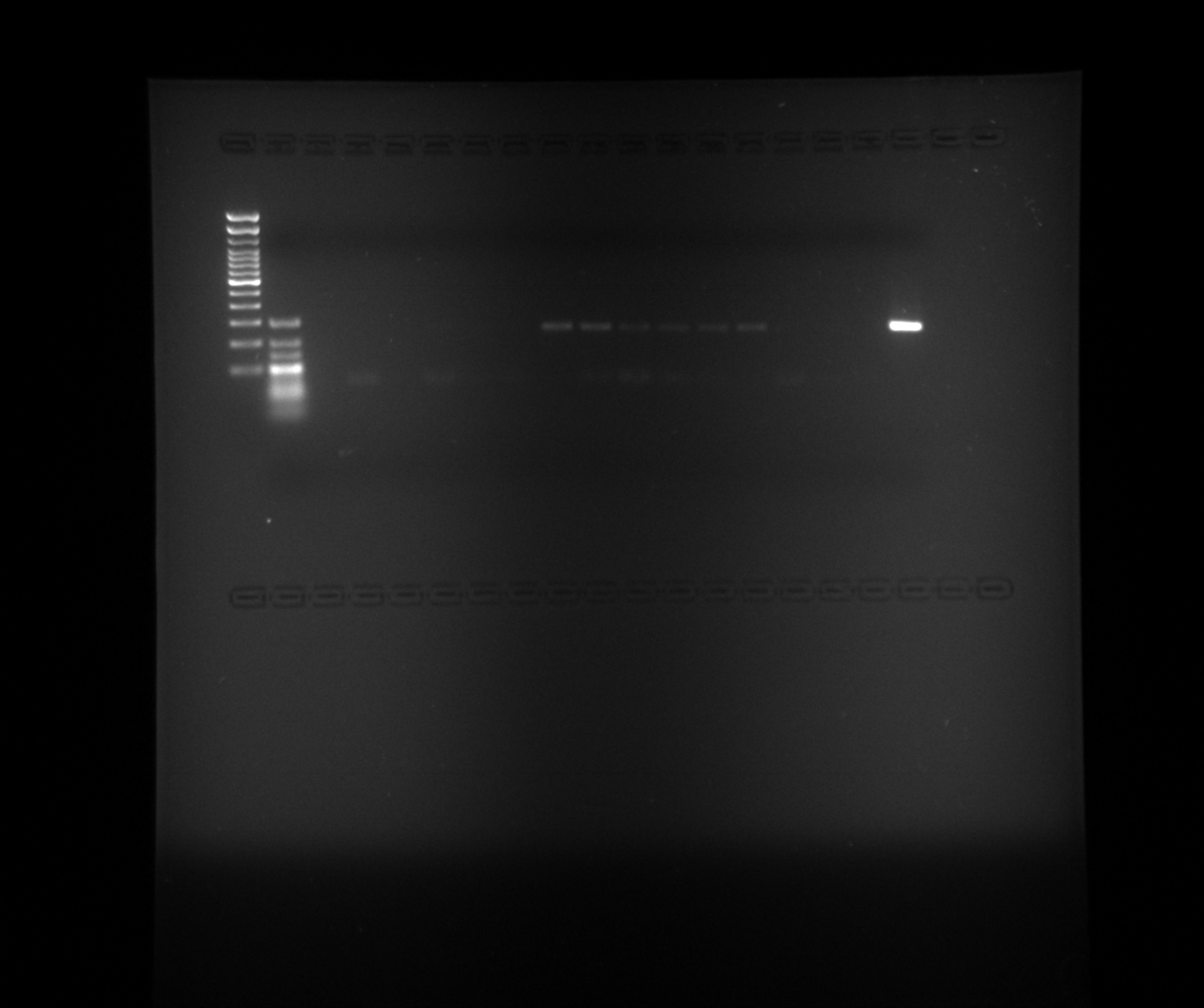

Supplement: Source data 1. — Source data for sequences and plasmid creation in detail are provided in a ZIP file called “Source data Sequences and Plasmid creation”. The source data of FACS for Figure 4 and its supplements is included in a Microsoft Excel spreadsheet called “Source data - FACS”. [file elife-72638-data1.zip › Source data Gels/Figure 3-source data 2-panel E-L1.tif]

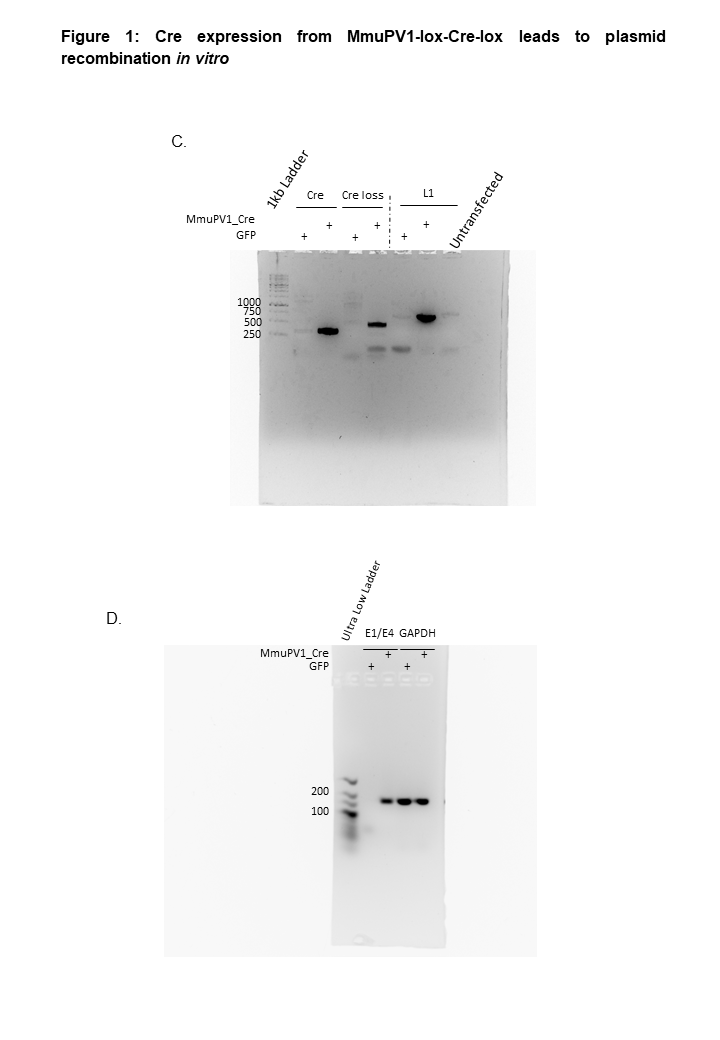

Supplement: Source data 1. — Source data for sequences and plasmid creation in detail are provided in a ZIP file called “Source data Sequences and Plasmid creation”. The source data of FACS for Figure 4 and its supplements is included in a Microsoft Excel spreadsheet called “Source data - FACS”. [file elife-72638-data1.zip › Source data Gels/Figure 1-source data 1-labelled.TIF]

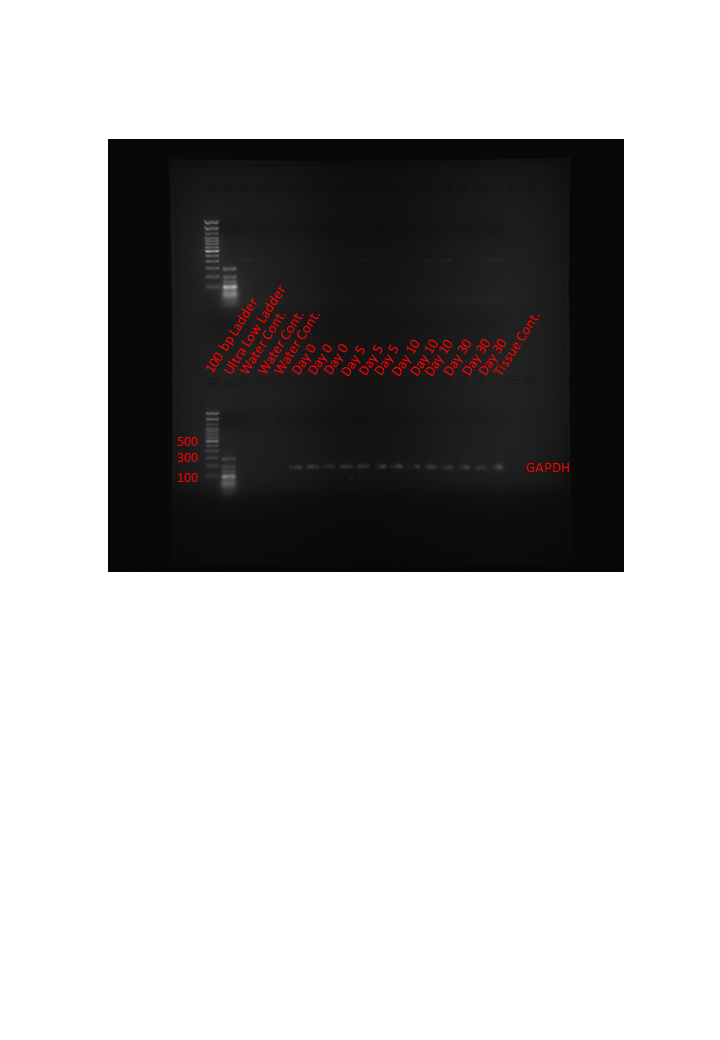

Supplement: Source data 1. — Source data for sequences and plasmid creation in detail are provided in a ZIP file called “Source data Sequences and Plasmid creation”. The source data of FACS for Figure 4 and its supplements is included in a Microsoft Excel spreadsheet called “Source data - FACS”. [file elife-72638-data1.zip › Source data Gels/Figure 3-source data 2-labelled.TIF]

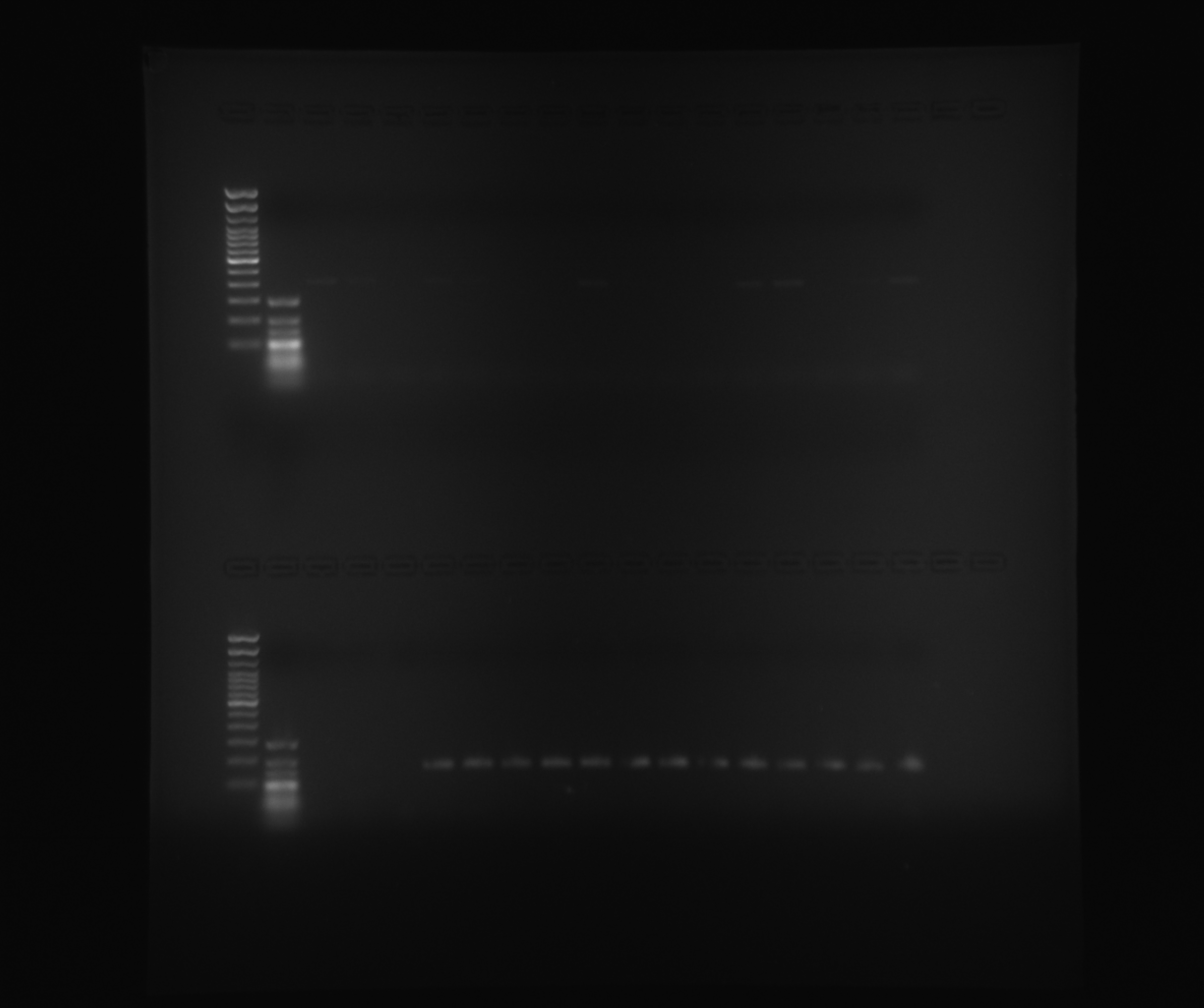

Supplement: Source data 1. — Source data for sequences and plasmid creation in detail are provided in a ZIP file called “Source data Sequences and Plasmid creation”. The source data of FACS for Figure 4 and its supplements is included in a Microsoft Excel spreadsheet called “Source data - FACS”. [file elife-72638-data1.zip › Source data Gels/Figure 3-source data 3-panel E-GAPDH.tif]

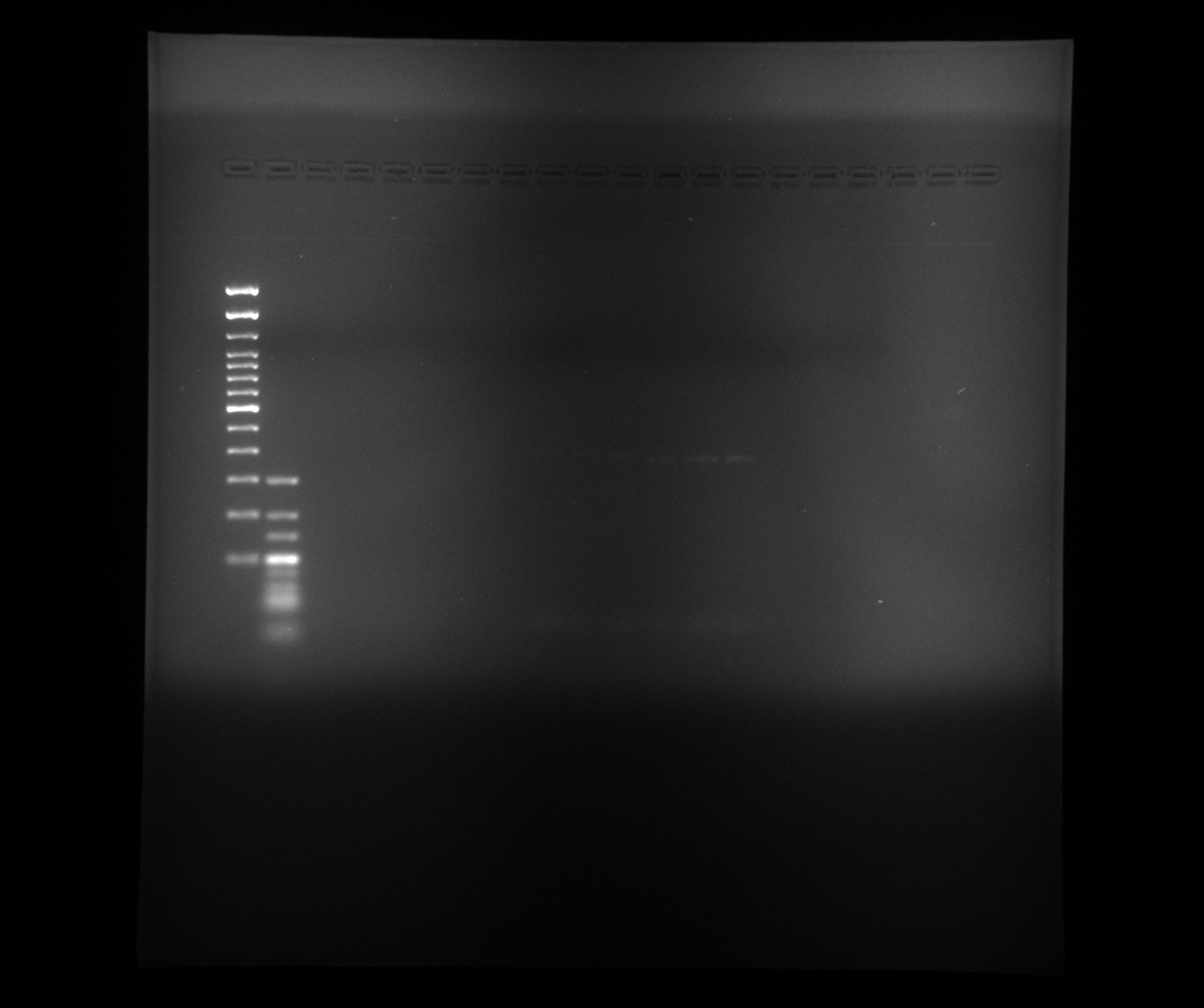

Supplement: Source data 1. — Source data for sequences and plasmid creation in detail are provided in a ZIP file called “Source data Sequences and Plasmid creation”. The source data of FACS for Figure 4 and its supplements is included in a Microsoft Excel spreadsheet called “Source data - FACS”. [file elife-72638-data1.zip › Source data Gels/Figure 3-source data 1-panel E-Cre-loss.tif]

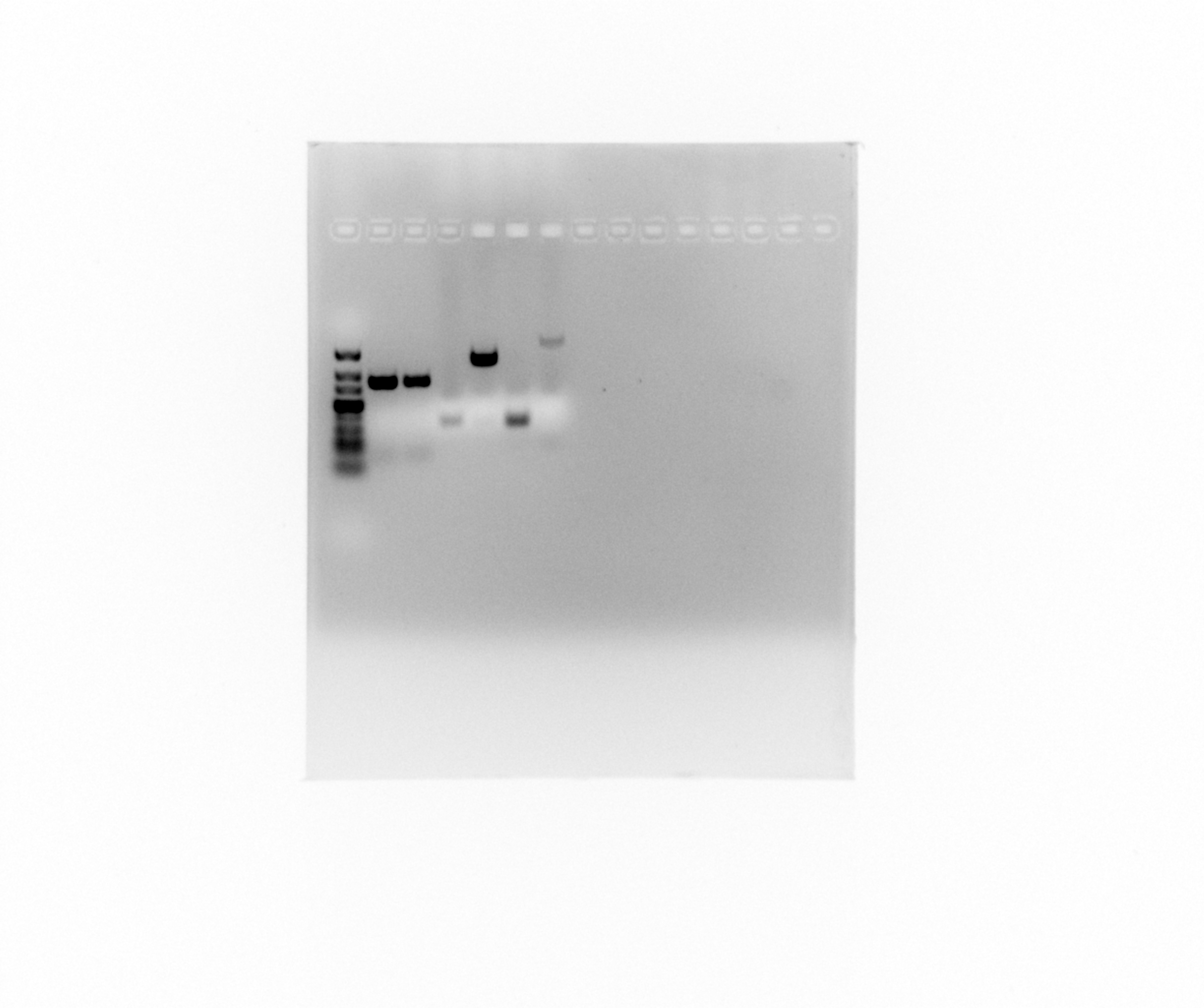

Supplement: Source data 1. — Source data for sequences and plasmid creation in detail are provided in a ZIP file called “Source data Sequences and Plasmid creation”. The source data of FACS for Figure 4 and its supplements is included in a Microsoft Excel spreadsheet called “Source data - FACS”. [file elife-72638-data1.zip › Source data Gels/Figure 1-source data 3-panel E.tif]

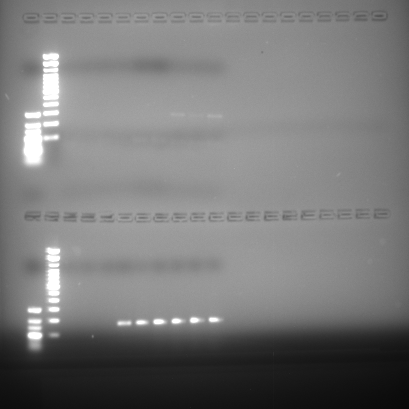

Supplement: Source data 1. — Source data for sequences and plasmid creation in detail are provided in a ZIP file called “Source data Sequences and Plasmid creation”. The source data of FACS for Figure 4 and its supplements is included in a Microsoft Excel spreadsheet called “Source data - FACS”. [file elife-72638-data1.zip › Source data Gels/Figure 3 suppl1-source data 2-panel C GAPDH and MmuPV1-L1.TIF]

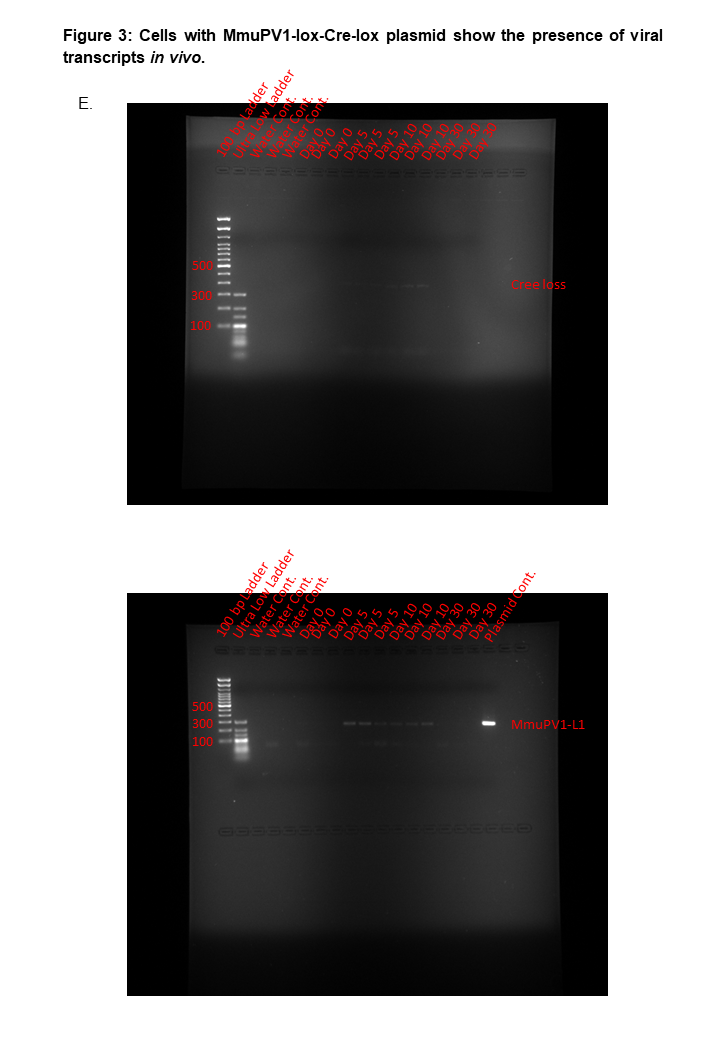

Supplement: Source data 1. — Source data for sequences and plasmid creation in detail are provided in a ZIP file called “Source data Sequences and Plasmid creation”. The source data of FACS for Figure 4 and its supplements is included in a Microsoft Excel spreadsheet called “Source data - FACS”. [file elife-72638-data1.zip › Source data Gels/Figure 3-source data 1-labelled.TIF]

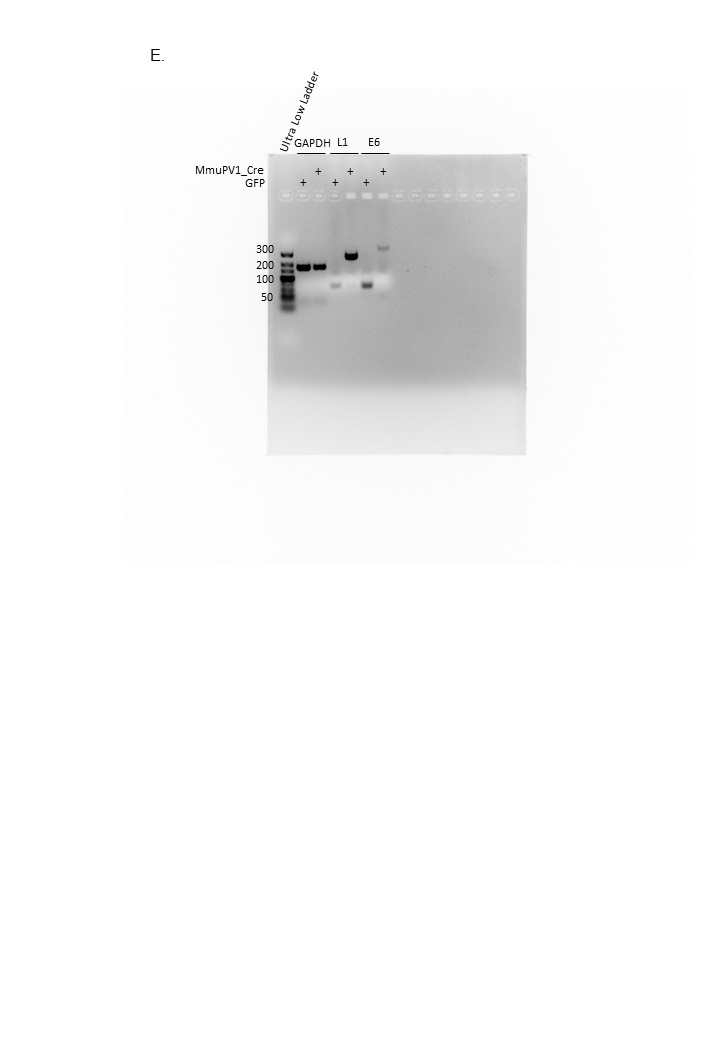

Supplement: Source data 1. — Source data for sequences and plasmid creation in detail are provided in a ZIP file called “Source data Sequences and Plasmid creation”. The source data of FACS for Figure 4 and its supplements is included in a Microsoft Excel spreadsheet called “Source data - FACS”. [file elife-72638-data1.zip › Source data Gels/Figure 1-source data 2-labelled.TIF]

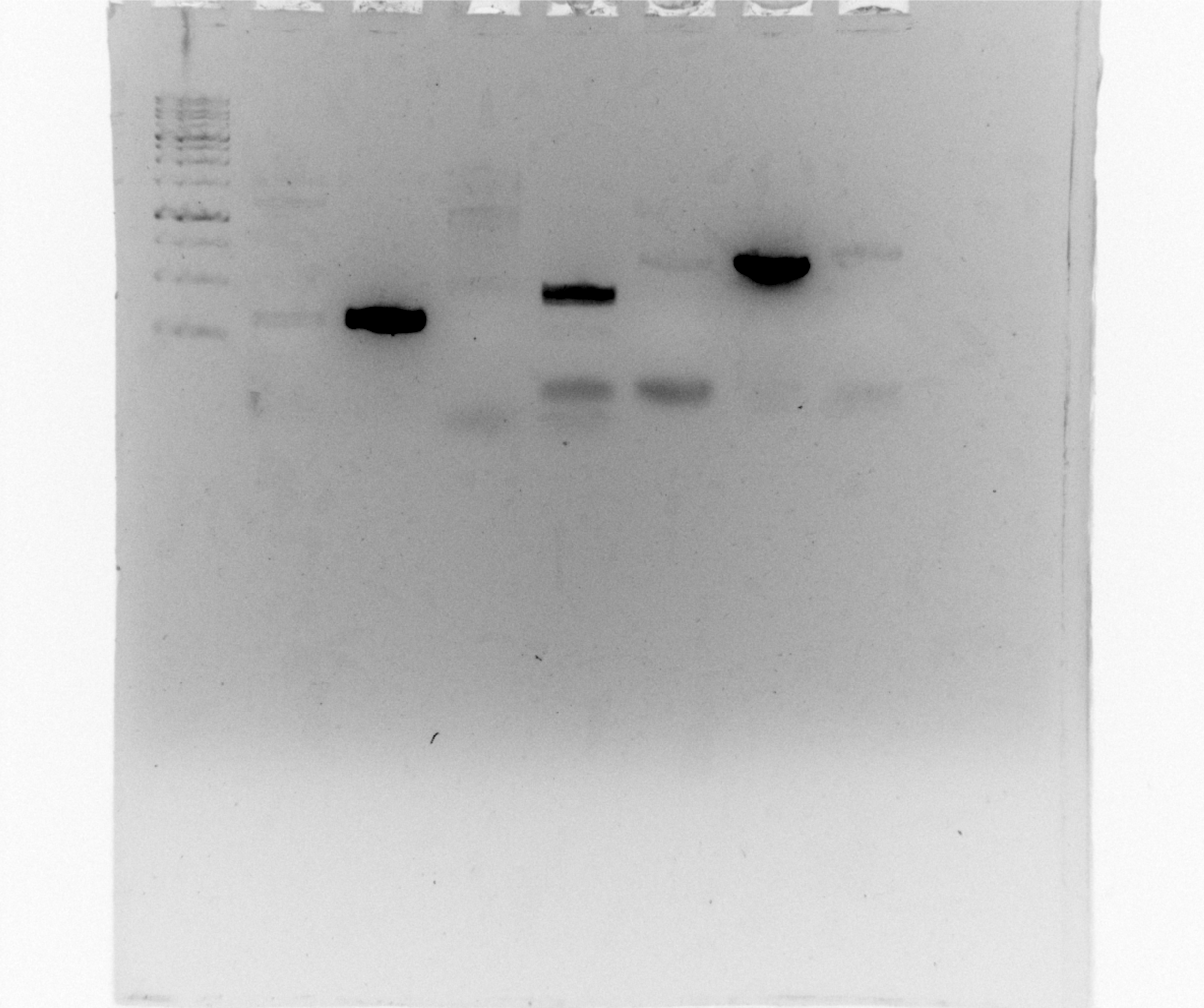

Supplement: Source data 1. — Source data for sequences and plasmid creation in detail are provided in a ZIP file called “Source data Sequences and Plasmid creation”. The source data of FACS for Figure 4 and its supplements is included in a Microsoft Excel spreadsheet called “Source data - FACS”. [file elife-72638-data1.zip › Source data Gels/Figure 1-source data 1-panel C.tif]

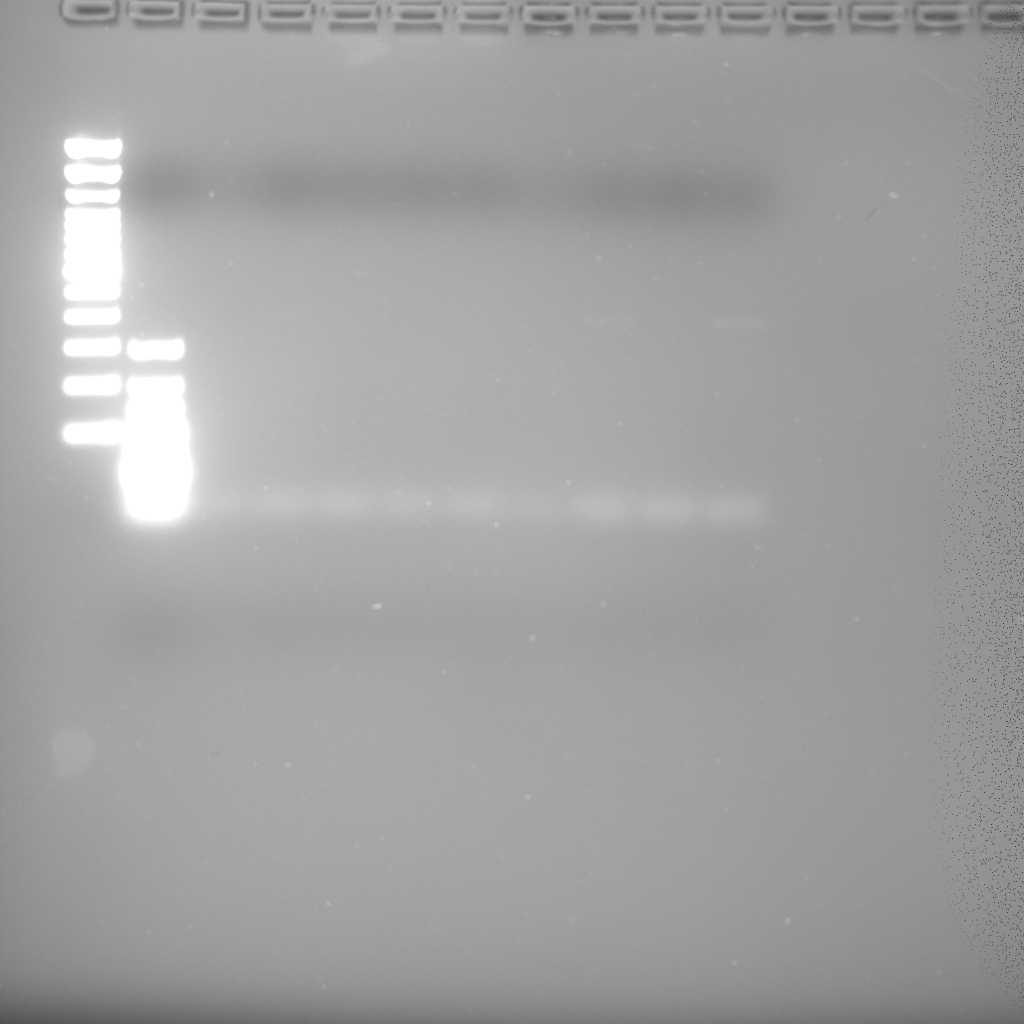

Supplement: Source data 1. — Source data for sequences and plasmid creation in detail are provided in a ZIP file called “Source data Sequences and Plasmid creation”. The source data of FACS for Figure 4 and its supplements is included in a Microsoft Excel spreadsheet called “Source data - FACS”. [file elife-72638-data1.zip › Source data Gels/Figure 3 suppl 1-source data 1-panel C Cre loss.TIF]

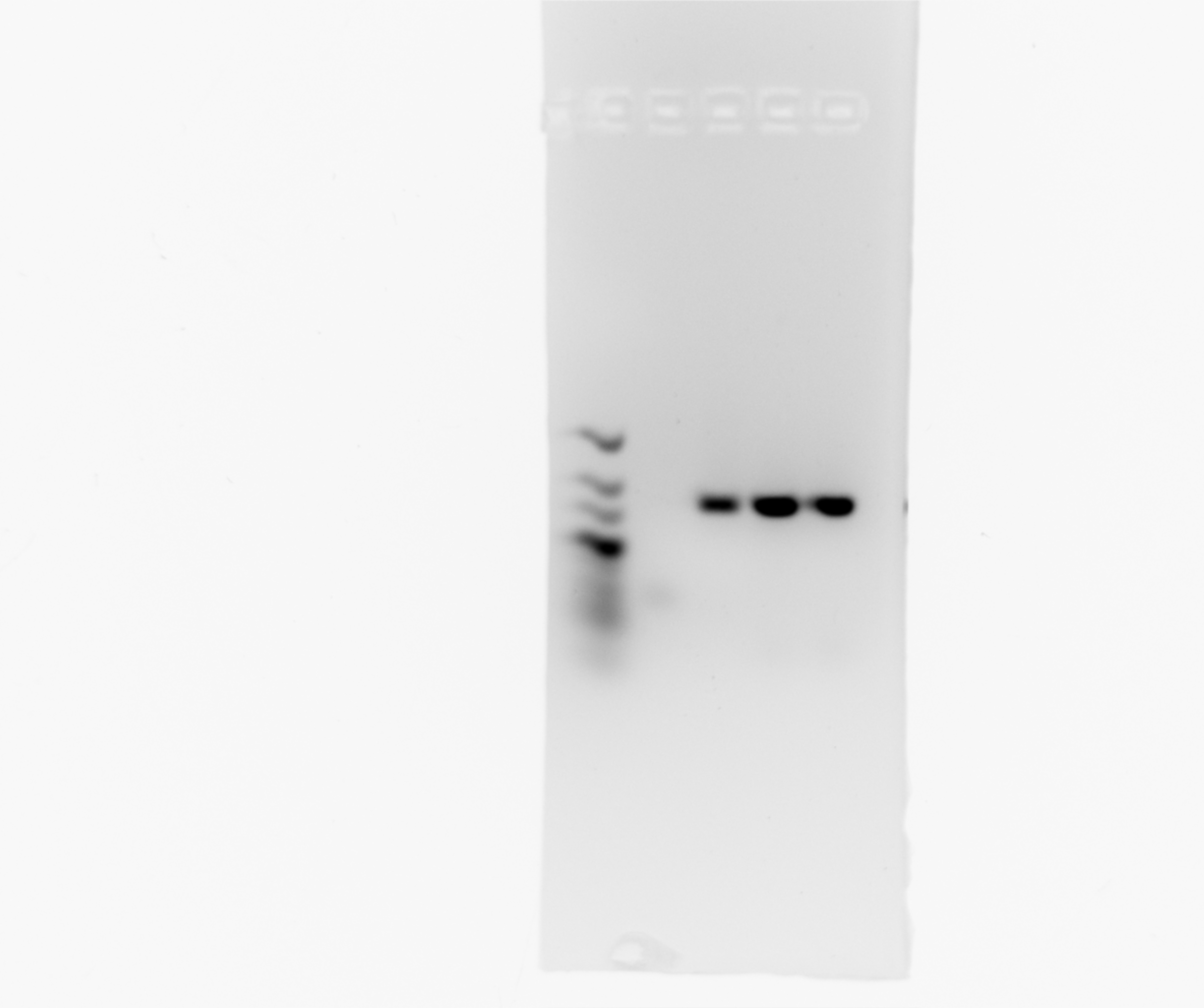

Supplement: Source data 1. — Source data for sequences and plasmid creation in detail are provided in a ZIP file called “Source data Sequences and Plasmid creation”. The source data of FACS for Figure 4 and its supplements is included in a Microsoft Excel spreadsheet called “Source data - FACS”. [file elife-72638-data1.zip › Source data Gels/Figure 1-source data 2-panel D.tif]

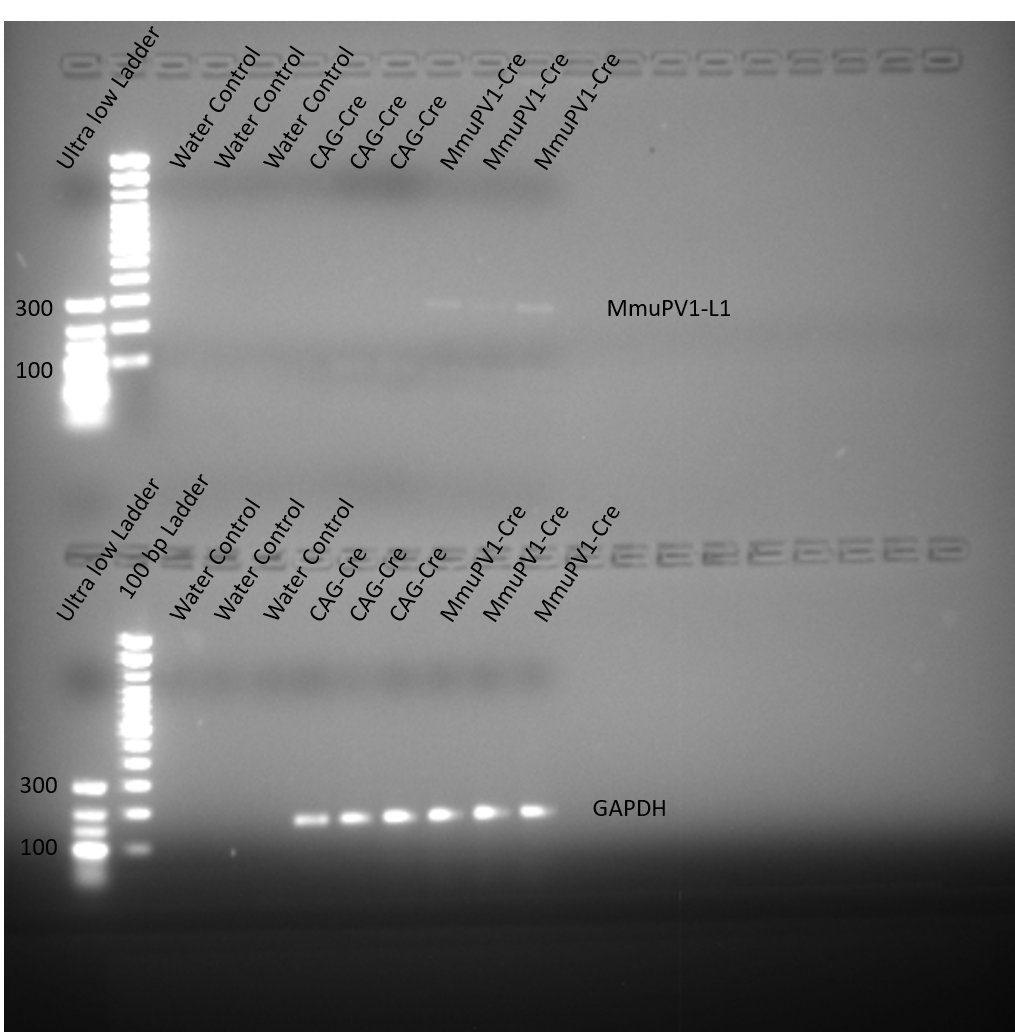

Supplement: Source data 1. — Source data for sequences and plasmid creation in detail are provided in a ZIP file called “Source data Sequences and Plasmid creation”. The source data of FACS for Figure 4 and its supplements is included in a Microsoft Excel spreadsheet called “Source data - FACS”. [file elife-72638-data1.zip › Source data Gels/Figure 3 suppl 1-source data 2-panel C GAPDH and MmuPV1-L1-labelled.tif]

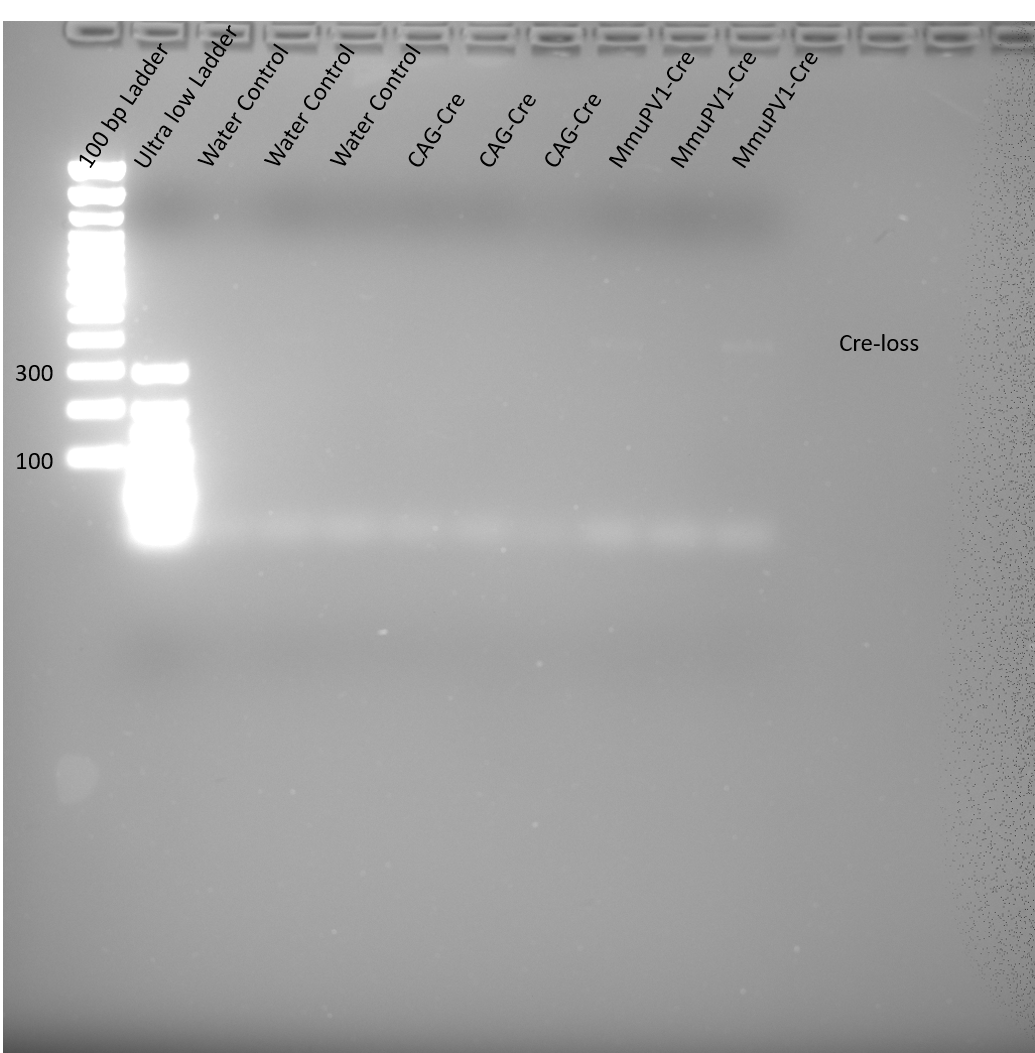

Supplement: Source data 1. — Source data for sequences and plasmid creation in detail are provided in a ZIP file called “Source data Sequences and Plasmid creation”. The source data of FACS for Figure 4 and its supplements is included in a Microsoft Excel spreadsheet called “Source data - FACS”. [file elife-72638-data1.zip › Source data Gels/Figure 3 suppl 1-source data 1-panel C Cre loss-labelled.tif]

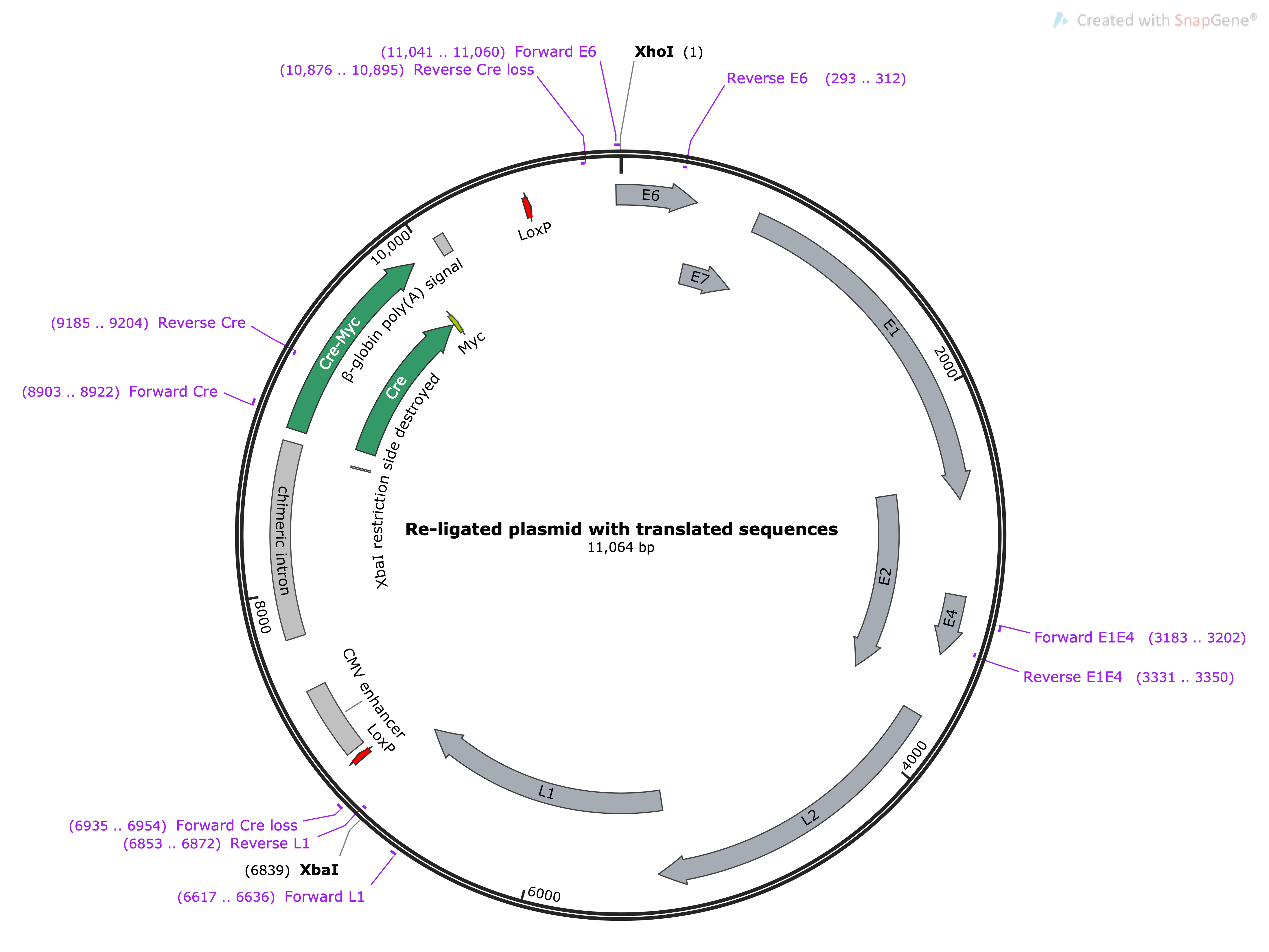

Supplement: Source data 2. [file elife-72638-data2.zip › Sequences and Plasmid creation/Re-ligated plasmid with translated sequences Map.tiff]
