# Supplementary material for: A novel lineage-tracing mouse model for studying early MmuPV1 infections
Source: eLife. 2022 May 9;11:e72638. doi: 10.7554/eLife.72638 (PMC9084889; doi:10.7554/eLife.72638)
Supplement: Source data 2. [file elife-72638-data2.zip › Sequences and Plasmid creation/MmuPV1_lox-Cre-loxst sequences of steps.docx]

1. PCR product -large-lox (PstI-HindIII)

ctgcagCCAGTGAGCGCGACGTAATACGACTCACTATAGGGCGAATTGGCGGAAGGCCGTCAAGGCCACGTGTCTTGTCCAGAGCTCGGGAACGGTGAATTCATAACTTCGTATAGCATACATTATACGAAGTTACCAGTGTGCCTCGCCTCATTCTTTAGCTCGCACCTGGGCTCACTTTGTGCCAGACTGTCATAACAAACAGTCTCTGTTGGCTGTGTGCTCTCTAATTTCTCGAAAAGACGTGTTTTGACGAAGGACCGTTTTCGGTCGGGCGCCAGTATCAGCATAAACTCCAGCCAATTTGGCCAAGGTAAGGAAATGACTAACTGTCTTGGAACAGATGCGTGTCCTGGCAATTATCCGCGTACCGTTTTCGGTCGGGTAAAAAAGGCGCCAAGCTAAGCATGATTCAGAGTTCCATTGTGTTCTGCCAAGTACAGGTGTGGTGTTCTGGAACGGTCGTACAATTAATCTTTGAGCTGATGGTTGGCAACAATTATTTCCCTCTGAAAAAATTTAGGTGGAGCGGGAACGGTCGCATATAAGTATCAGTGTGCCCCCATAACCGTATTCGTTCATGGAAATCGGCAAAGGCTACACT***CTCGAG***GGGAACGGTGGTACCTGGAGCACAAGACTGGCCTCATGaagctt

1. Clone in pCre between PstI and HindIII – this is confirmed by sequencing
2. Digest Small-lox sequence from Small plasmid (SacI and KpnI)

GAGCTCGATAACGGTCCGCGGGGTCAAAAGGGCAGCGTCTACAACCTCTGCGTCT***TCTAGA***CGAGTTGTAAAACGGAAGAGGGGAAGCAAATAACTGAACTGGTGCTACTAACTGAATGACTCCGGTATTATGAAGTTCTTGTATTGTATAACTGTTTACTGGGGGCTTACTGTGTATAGGGGGCTTGAGTTGTTTGTCTGTTCTTGTCCATGTCCTTGTGATGTACTTTTGCAACTTAAATAAATGACTAATGCTGAATAACTTCGTATAGCATACATTATACGAAGTTATGAATTCGGGAACGGTATATGAACGGTGGTACC

SacI and KpnI after blunting

CGATAACGGTCCGCGGGGTCAAAAGGGCAGCGTCTACAACCTCTGCGTCT***TCTAGA***CGAGTTGTAAAACGGAAGAGGGGAAGCAAATAACTGAACTGGTGCTACTAACTGAATGACTCCGGTATTATGAAGTTCTTGTATTGTATAACTGTTTACTGGGGGCTTACTGTGTATAGGGGGCTTGAGTTGTTTGTCTGTTCTTGTCCATGTCCTTGTGATGTACTTTTGCAACTTAAATAAATGACTAATGCTGAATAACTTCGTATAGCATACATTATACGAAGTTATGAATTCGGGAACGGTATATGAACGGTG

1. Blunt the ends and clone in plasmid from step 2 (digest plasmid from step 2 with SalI (GTCGAC) and create blunt ends) SalI after blunting (…GTCGA TCGAC…) ( this is confirmed by sequencing)

>step1 and step 2

ggtcgacattgattattgactagttattaatagtaatcaattacggggtcattagttcatagcccatatatggagttccgcgttacataacttacggtaaatggcccgcctggctgaccgcccaacgacccccgcccattgacgtcaataatgacgtatgttcccatagtaacgccaatagggactttccattgacgtcaatgggtggagtatttacggtaaactgcccacttggcagtacatcaagtgtatcatatgccaagtacgccccctattgacgtcaatgacggtaaatggcccgcctggcattatgcccagtacatgaccttatgggactttcctacttggcagtacatctacgtattagtcatcgctattaccatggtcgaggtgagccccacgttctgcttcactctccccatctcccccccctccccacccccaattttgtatttatttattttttaattattttgtgcagcgatgggggcggggggggggggggcgcgcgccaggcggggggggggggggggggggggggggggggggggggcgggggggggcggcggcagccaatcagagcggcgcgctccgaaagtttccttttatggcgaggcggcggcggcggcggccctataaaaagcgaagcgcgcggcgggcgggagtcgctgcgcgctgccttcgccccgtgccccgctccgccgccgcctcgcgccgcccgccccggctctgactgaccgcgttactcccacaggtgagcgggcgggacggcccttctcctccgggctgtaattagcgcttggtttaatgacggcttgtttcttttctgtggctgcgtgaaagccttgaggggctccgggagggccctttgtgcggggggagcggctcggggggtgcgtgcgtgtgtgtgtgcgtggggagcgccgcgtgcggctccgcgctgcccggcggctgtgagcgctgcgggcgcggcgcggggctttgtgcgctccgcagtgtgcgcgaggggagcgcggccgggggcggtgccccgcggtgcggggggggctgcgaggggaacaaaggctgcgtgcggggtgtgtgcgtgggggggtgagcagggggtgtgggcgcgtcggtcgggctgcaaccccccctgcacccccctccccgagttgctgagcacggcccggcttcgggtgcggggctccgtacggggcgtggcgcggggctcgccgtgccgggcggggggtggcggcaggtgggggtgccgggcggggcggggccgcctcgggccggggagggctcgggggaggggcgcggcggcccccggagcgccggcggctgtcgaggcgcggcgagccgcagccattgccttttatggtaatcgtgcgagagggcgcagggacttcctttgtcccaaatctgtgcggagccgaaatctgggaggcgccgccgcaccccctctagcgggcgcggggcgaagcggtgcggcgccggcaggaaggaaatgggcggggagggccttcgtgcgtcgccgcgccgccgtccccttctccctctccagcctcggggctgtccgcggggggacggctgccttcgggggggacggggcagggcggggttcggcttctggcgtgtgaccggcggc***TCTAGA***gcctctgctaaccatgttcatgccttcttctttttcctacagctcctgggcaacgtgctggttattgtgctgtctcatcattttggcaaagaattctgagccgccaccatggccaatttactgaccgtacaccaaaatttgcctgcattaccggtcgatgcaacgagtgatgaggttcgcaagaacctgatggacatgttcagggatcgccaggcgttttctgagcatacctggaaaatgcttctgtccgtttgccggtcgtgggcggcatggtgcaagttgaataaccggaaatggtttcccgcagaacctgaagatgttcgcgattatcttctatatcttcaggcgcgcggtctggcagtaaaaactatccagcaacatttgggccagctaaacatgcttcatcgtcggtccgggctgccacgaccaagtgacagcaatgctgtttcactggttatgcggcggatccgaaaagaaaacgttgatgccggtgaacgtgcaaaacaggctctagcgttcgaacgcactgatttcgaccaggttcgttcactcatggaaaatagcgatcgctgccaggatatacgtaatctggcatttctggggattgcttataacaccctgttacgtatagccgaaattgccaggatcagggttaaagatatctcacgtactgacggtgggagaatgttaatccatattggcagaacgaaaacgctggttagcaccgcaggtgtagagaaggcacttagcctgggggtaactaaactggtcgagcgatggatttccgtctctggtgtagctgatgatccgaataactacctgttttgccgggtcagaaaaaatggtgttgccgcgccatctgccaccagccagctatcaactcgcgccctggaagggatttttgaagcaactcatcgattgatttacggcgctaaggatgactctggtcagagatacctggcctggtctggacacagtgcccgtgtcggagccgcgcgagatatggcccgcgctggagtttcaataccggagatcatgcaagctggtggctggaccaatgtaaatattgtcatgaactatatccgtaacctggatagtgaaacaggggcaatggtgcgcctgctggaagatggcgatggaccggtggaacaaaaacttatttctgaagaagatctgtgatagcggccgcactcctcaggtgcaggctgcctatcagaaggtggtggctggtgtggccaatgccctggctcacaaataccactgagatctttttccctctgccaaaaattatggggacatcatgaagccccttgagcatctgacttctggctaataaaggaaatttattttcattgcaatagtgtgttggaattttttgtgtctctcactcggaaggacatatgggagggcaaatcatttaaaacatcagaatgagtatttggtttagagtttggcaacatatgcccatatgctggctgccatgaacaaaggttggctataaagaggtcatcagtatatgaaacagccccctgctgtccattccttattccatagaaaagccttgacttgaggttagattttttttatattttgttttgtgttatttttttctttaacatccctaaaattttccttacatgttttactagccagatttttcctcctctcctgactactcccagtcatagctgtccctcttctcttatggagatccctcgacctgcagCCAGTGAGCGCGACGTAATACGACTCACTATAGGGCGAATTGGCGGAAGGCCGTCAAGGCCACGTGTCTTGTCCAGAGCTCGGGAACGGTGAATTCATAACTTCGTATAGCATACATTATACGAAGTTACCAGTGTGCCTCGCCTCATTCTTTAGCTCGCACCTGGGCTCACTTTGTGCCAGACTGTCATAACAAACAGTCTCTGTTGGCTGTGTGCTCTCTAATTTCTCGAAAAGACGTGTTTTGACGAAGGACCGTTTTCGGTCGGGCGCCAGTATCAGCATAAACTCCAGCCAATTTGGCCAAGGTAAGGAAATGACTAACTGTCTTGGAACAGATGCGTGTCCTGGCAATTATCCGCGTACCGTTTTCGGTCGGGTAAAAAAGGCGCCAAGCTAAGCATGATTCAGAGTTCCATTGTGTTCTGCCAAGTACAGGTGTGGTGTTCTGGAACGGTCGTACAATTAATCTTTGAGCTGATGGTTGGCAACAATTATTTCCCTCTGAAAAAATTTAGGTGGAGCGGGAACGGTCGCATATAAGTATCAGTGTGCCCCCATAACCGTATTCGTTCATGGAAATCGGCAAAGGCTACACT***CTCGAG***GGGAACGGTGGTACCtgGAGCACAAGACTGGCCTCATGaagcttggcgtaatcatggtcatagctgtttcctgtgtgaaattgttatccgctcacaattccacacaacatacgagccggaagcataaagtgtaaagcctggggtgcctaatgagtgagctaactcacattaattgcgttgcgctcactgcccgctttccagtcgggaaacctgtcgtgccagcggatccgcatctcaattagtcagcaaccatagtcccgcccctaactccgcccatcccgcccctaactccgcccagttccgcccattctccgccccatggctgactaattttttttatttatgcagaggccgaggccgcctcggcctctgagctattccagaagtagtgaggaggcttttttggaggcctaggcttttgcaaaaagctaacttgtttattgcagcttataatggttacaaataaagcaatagcatcacaaatttcacaaataaagcatttttttcactgcattctagttgtggtttgtccaaactcatcaatgtatcttatcatgtctggatccgctgcattaatgaatcggccaacgcgcggggagaggcggtttgcgtattgggcgctcttccgcttcctcgctcactgactcgctgcgctcggtcgttcggctgcggcgagcggtatcagctcactcaaaggcggtaatacggttatccacagaatcaggggataacgcaggaaagaacatgtgagcaaaaggccagcaaaaggccaggaaccgtaaaaaggccgcgttgctggcgtttttccataggctccgcccccctgacgagcatcacaaaaatcgacgctcaagtcagaggtggcgaaacccgacaggactataaagataccaggcgtttccccctggaagctccctcgtgcgctctcctgttccgaccctgccgcttaccggatacctgtccgcctttctcccttcgggaagcgtggcgctttctcatagctcacgctgtaggtatctcagttcggtgtaggtcgttcgctccaagctgggctgtgtgcacgaaccccccgttcagcccgaccgctgcgccttatccggtaactatcgtcttgagtccaacccggtaagacacgacttatcgccactggcagcagccactggtaacaggattagcagagcgaggtatgtaggcggtgctacagagttcttgaagtggtggcctaactacggctacactagaagaacagtatttggtatctgcgctctgctgaagccagttaccttcggaaaaagagttggtagctcttgatccggcaaacaaaccaccgctggtagcggtggtttttttgtttgcaagcagcagattacgcgcagaaaaaaaggatctcaagaagatcctttgatcttttctacggggtctgacgctcagtggaacgaaaactcacgttaagggattttggtcatgagattatcaaaaaggatcttcacctagatccttttaaattaaaaatgaagttttaaatcaatctaaagtatatatgagtaaacttggtctgacagttaccaatgcttaatcagtgaggcacctatctcagcgatctgtctatttcgttcatccatagttgcctgactccccgtcgtgtagataactacgatacgggagggcttaccatctggccccagtgctgcaatgataccgcgagacccacgctcaccggctccagatttatcagcaataaaccagccagccggaagggccgagcgcagaagtggtcctgcaactttatccgcctccatccagtctattaattgttgccgggaagctagagtaagtagttcgccagttaatagtttgcgcaacgttgttgccattgctacaggcatcgtggtgtcacgctcgtcgtttggtatggcttcattcagctccggttcccaacgatcaaggcgagttacatgatcccccatgttgtgcaaaaaagcggttagctccttcggtcctccgatcgttgtcagaagtaagttggccgcagtgttatcactcatggttatggcagcactgcataattctcttactgtcatgccatccgtaagatgcttttctgtgactggtgagtactcaaccaagtcattctgagaatagtgtatgcggcgaccgagttgctcttgcccggcgtcaatacgggataataccgcgccacatagcagaactttaaaagtgctcatcattggaaaacgttcttcggggcgaaaactctcaaggatcttaccgctgttgagatccagttcgatgtaacccactcgtgcacccaactgatcttcagcatcttttactttcaccagcgtttctgggtgagcaaaaacaggaaggcaaaatgccgcaaaaaagggaataagggcgacacggaaatgttgaatactcatactcttcctttttcaatattattgaagcatttatcagggttattgtctcatgagcggatacatatttgaatgtatttagaaaaataaacaaataggggttccgcgcacatttccccgaaaagtgccacctg

>step3 and step 4

>lox-cre-lox plasmid (including small and large plasmid sequences)

gtcgaCGATAACGGTCCGCGGGGTCAAAAGGGCAGCGTCTACAACCTCTGCGTCT***TCTAGA***CGAGTTGTAAAACGGAAGAGGGGAAGCAAATAACTGAACTGGTGCTACTAACTGAATGACTCCGGTATTATGAAGTTCTTGTATTGTATAACTGTTTACTGGGGGCTTACTGTGTATAGGGGGCTTGAGTTGTTTGTCTGTTCTTGTCCATGTCCTTGTGATGTACTTTTGCAACTTAAATAAATGACTAATGCTGAATAACTTCGTATAGCATACATTATACGAAGTTATGAATTCGGGAACGGTATATGAACGGTGtcgacattgattattgactagttattaatagtaatcaattacggggtcattagttcatagcccatatatggagttccgcgttacataacttacggtaaatggcccgcctggctgaccgcccaacgacccccgcccattgacgtcaataatgacgtatgttcccatagtaacgccaatagggactttccattgacgtcaatgggtggagtatttacggtaaactgcccacttggcagtacatcaagtgtatcatatgccaagtacgccccctattgacgtcaatgacggtaaatggcccgcctggcattatgcccagtacatgaccttatgggactttcctacttggcagtacatctacgtattagtcatcgctattaccatggtcgaggtgagccccacgttctgcttcactctccccatctcccccccctccccacccccaattttgtatttatttattttttaattattttgtgcagcgatgggggcggggggggggggggcgcgcgccaggcggggggggggggggggggggggggggggggggggggcgggggggggcggcggcagccaatcagagcggcgcgctccgaaagtttccttttatggcgaggcggcggcggcggcggccctataaaaagcgaagcgcgcggcgggcgggagtcgctgcgcgctgccttcgccccgtgccccgctccgccgccgcctcgcgccgcccgccccggctctgactgaccgcgttactcccacaggtgagcgggcgggacggcccttctcctccgggctgtaattagcgcttggtttaatgacggcttgtttcttttctgtggctgcgtgaaagccttgaggggctccgggagggccctttgtgcggggggagcggctcggggggtgcgtgcgtgtgtgtgtgcgtggggagcgccgcgtgcggctccgcgctgcccggcggctgtgagcgctgcgggcgcggcgcggggctttgtgcgctccgcagtgtgcgcgaggggagcgcggccgggggcggtgccccgcggtgcggggggggctgcgaggggaacaaaggctgcgtgcggggtgtgtgcgtgggggggtgagcagggggtgtgggcgcgtcggtcgggctgcaaccccccctgcacccccctccccgagttgctgagcacggcccggcttcgggtgcggggctccgtacggggcgtggcgcggggctcgccgtgccgggcggggggtggcggcaggtgggggtgccgggcggggcggggccgcctcgggccggggagggctcgggggaggggcgcggcggcccccggagcgccggcggctgtcgaggcgcggcgagccgcagccattgccttttatggtaatcgtgcgagagggcgcagggacttcctttgtcccaaatctgtgcggagccgaaatctgggaggcgccgccgcaccccctctagcgggcgcggggcgaagcggtgcggcgccggcaggaaggaaatgggcggggagggccttcgtgcgtcgccgcgccgccgtccccttctccctctccagcctcggggctgtccgcggggggacggctgccttcgggggggacggggcagggcggggttcggcttctggcgtgtgaccggcggctctagctagagcctctgctaaccatgttcatgccttcttctttttcctacagctcctgggcaacgtgctggttattgtgctgtctcatcattttggcaaagaattctgagccgccaccatggccaatttactgaccgtacaccaaaatttgcctgcattaccggtcgatgcaacgagtgatgaggttcgcaagaacctgatggacatgttcagggatcgccaggcgttttctgagcatacctggaaaatgcttctgtccgtttgccggtcgtgggcggcatggtgcaagttgaataaccggaaatggtttcccgcagaacctgaagatgttcgcgattatcttctatatcttcaggcgcgcggtctggcagtaaaaactatccagcaacatttgggccagctaaacatgcttcatcgtcggtccgggctgccacgaccaagtgacagcaatgctgtttcactggttatgcggcggatccgaaaagaaaacgttgatgccggtgaacgtgcaaaacaggctctagcgttcgaacgcactgatttcgaccaggttcgttcactcatggaaaatagcgatcgctgccaggatatacgtaatctggcatttctggggattgcttataacaccctgttacgtatagccgaaattgccaggatcagggttaaagatatctcacgtactgacggtgggagaatgttaatccatattggcagaacgaaaacgctggttagcaccgcaggtgtagagaaggcacttagcctgggggtaactaaactggtcgagcgatggatttccgtctctggtgtagctgatgatccgaataactacctgttttgccgggtcagaaaaaatggtgttgccgcgccatctgccaccagccagctatcaactcgcgccctggaagggatttttgaagcaactcatcgattgatttacggcgctaaggatgactctggtcagagatacctggcctggtctggacacagtgcccgtgtcggagccgcgcgagatatggcccgcgctggagtttcaataccggagatcatgcaagctggtggctggaccaatgtaaatattgtcatgaactatatccgtaacctggatagtgaaacaggggcaatggtgcgcctgctggaagatggcgatggaccggtggaacaaaaacttatttctgaagaagatctgtgatagcggccgcactcctcaggtgcaggctgcctatcagaaggtggtggctggtgtggccaatgccctggctcacaaataccactgagatctttttccctctgccaaaaattatggggacatcatgaagccccttgagcatctgacttctggctaataaaggaaatttattttcattgcaatagtgtgttggaattttttgtgtctctcactcggaaggacatatgggagggcaaatcatttaaaacatcagaatgagtatttggtttagagtttggcaacatatgcccatatgctggctgccatgaacaaaggttggctataaagaggtcatcagtatatgaaacagccccctgctgtccattccttattccatagaaaagccttgacttgaggttagattttttttatattttgttttgtgttatttttttctttaacatccctaaaattttccttacatgttttactagccagatttttcctcctctcctgactactcccagtcatagctgtccctcttctcttatggagatccctcgacctgcagCCAGTGAGCGCGACGTAATACGACTCACTATAGGGCGAATTGGCGGAAGGCCGTCAAGGCCACGTGTCTTGTCCAGAGCTCGGGAACGGTGAATTCATAACTTCGTATAGCATACATTATACGAAGTTACCAGTGTGCCTCGCCTCATTCTTTAGCTCGCACCTGGGCTCACTTTGTGCCAGACTGTCATAACAAACAGTCTCTGTTGGCTGTGTGCTCTCTAATTTCTCGAAAAGACGTGTTTTGACGAAGGACCGTTTTCGGTCGGGCGCCAGTATCAGCATAAACTCCAGCCAATTTGGCCAAGGTAAGGAAATGACTAACTGTCTTGGAACAGATGCGTGTCCTGGCAATTATCCGCGTACCGTTTTCGGTCGGGTAAAAAAGGCGCCAAGCTAAGCATGATTCAGAGTTCCATTGTGTTCTGCCAAGTACAGGTGTGGTGTTCTGGAACGGTCGTACAATTAATCTTTGAGCTGATGGTTGGCAACAATTATTTCCCTCTGAAAAAATTTAGGTGGAGCGGGAACGGTCGCATATAAGTATCAGTGTGCCCCCATAACCGTATTCGTTCATGGAAATCGGCAAAGGCTACACT***CTCGAG***GGGAACGGTGGTACCtgGAGCACAAGACTGGCCTCATGaagcttggcgtaatcatggtcatagctgtttcctgtgtgaaattgttatccgctcacaattccacacaacatacgagccggaagcataaagtgtaaagcctggggtgcctaatgagtgagctaactcacattaattgcgttgcgctcactgcccgctttccagtcgggaaacctgtcgtgccagcggatccgcatctcaattagtcagcaaccatagtcccgcccctaactccgcccatcccgcccctaactccgcccagttccgcccattctccgccccatggctgactaattttttttatttatgcagaggccgaggccgcctcggcctctgagctattccagaagtagtgaggaggcttttttggaggcctaggcttttgcaaaaagctaacttgtttattgcagcttataatggttacaaataaagcaatagcatcacaaatttcacaaataaagcatttttttcactgcattctagttgtggtttgtccaaactcatcaatgtatcttatcatgtctggatccgctgcattaatgaatcggccaacgcgcggggagaggcggtttgcgtattgggcgctcttccgcttcctcgctcactgactcgctgcgctcggtcgttcggctgcggcgagcggtatcagctcactcaaaggcggtaatacggttatccacagaatcaggggataacgcaggaaagaacatgtgagcaaaaggccagcaaaaggccaggaaccgtaaaaaggccgcgttgctggcgtttttccataggctccgcccccctgacgagcatcacaaaaatcgacgctcaagtcagaggtggcgaaacccgacaggactataaagataccaggcgtttccccctggaagctccctcgtgcgctctcctgttccgaccctgccgcttaccggatacctgtccgcctttctcccttcgggaagcgtggcgctttctcatagctcacgctgtaggtatctcagttcggtgtaggtcgttcgctccaagctgggctgtgtgcacgaaccccccgttcagcccgaccgctgcgccttatccggtaactatcgtcttgagtccaacccggtaagacacgacttatcgccactggcagcagccactggtaacaggattagcagagcgaggtatgtaggcggtgctacagagttcttgaagtggtggcctaactacggctacactagaagaacagtatttggtatctgcgctctgctgaagccagttaccttcggaaaaagagttggtagctcttgatccggcaaacaaaccaccgctggtagcggtggtttttttgtttgcaagcagcagattacgcgcagaaaaaaaggatctcaagaagatcctttgatcttttctacggggtctgacgctcagtggaacgaaaactcacgttaagggattttggtcatgagattatcaaaaaggatcttcacctagatccttttaaattaaaaatgaagttttaaatcaatctaaagtatatatgagtaaacttggtctgacagttaccaatgcttaatcagtgaggcacctatctcagcgatctgtctatttcgttcatccatagttgcctgactccccgtcgtgtagataactacgatacgggagggcttaccatctggccccagtgctgcaatgataccgcgagacccacgctcaccggctccagatttatcagcaataaaccagccagccggaagggccgagcgcagaagtggtcctgcaactttatccgcctccatccagtctattaattgttgccgggaagctagagtaagtagttcgccagttaatagtttgcgcaacgttgttgccattgctacaggcatcgtggtgtcacgctcgtcgtttggtatggcttcattcagctccggttcccaacgatcaaggcgagttacatgatcccccatgttgtgcaaaaaagcggttagctccttcggtcctccgatcgttgtcagaagtaagttggccgcagtgttatcactcatggttatggcagcactgcataattctcttactgtcatgccatccgtaagatgcttttctgtgactggtgagtactcaaccaagtcattctgagaatagtgtatgcggcgaccgagttgctcttgcccggcgtcaatacgggataataccgcgccacatagcagaactttaaaagtgctcatcattggaaaacgttcttcggggcgaaaactctcaaggatcttaccgctgttgagatccagttcgatgtaacccactcgtgcacccaactgatcttcagcatcttttactttcaccagcgtttctgggtgagcaaaaacaggaaggcaaaatgccgcaaaaaagggaataagggcgacacggaaatgttgaatactcatactcttcctttttcaatattattgaagcatttatcagggttattgtctcatgagcggatacatatttgaatgtatttagaaaaataaacaaataggggttccgcgcacatttccccgaaaagtgccacctg

XbaI restriction side was modified by XbaI digestion followed by blunting with T4 DNA polymerase (TCTAGCTAGA) before cloning in the small and large fragments.

1. Digestion of MmupV1 and lox-cre-lox plasmid with ***XbaI*** and ***XhoI*** to remove Amp selection cassette and ligation of the two fragment.

MmupV1 fragment ***XhoI*** and ***XbaI*** digested

***CTCGAG***gaggtgcttagatattctaacaaagatgtcgtggattttcatttgtcttgtgctttttgctctactactatggatcataacgagaaggccagattcatacaggctaaattgaaatgtgttgttagagattttgcttttaaaggtgcttgtattgtgtgccgcagacagcttgcttgcaaggaaaagcttttgcatactagagttacaggggaggctgatttggtagagtgcatggctggcaagaatattgtgtttgttactgtaagatgtgttacgtgcctggcactccttactgcctctgaaaagcttgatgccaaagcgtgcggcttgccatttcacttggtgcgccacatgtggagaggctactgcgggttctgcaaaccattactataatgcagggcccattaccaacaattgctgacatcgagattcagaatctcgactcacttttgggtgttggtgagcctgacctacccgatgttgggtcatcatcgttgtcaccagactcgttaggagaagaggaggagctggagctggagactatcgatgtagatccttacaggattaaaacaacctgcttttgctgcgacactgttctccggttcataattgtgaccggagacgactcggtgaaagcattcgagtcactgcttctgcaggatcttagctttgtctgcccgcactgcgtcgcgtcgtacgtgaacctcagaaatggaaaacgataaaggtacagggcagtattctggatggtgttttatagataatgaggctgaatgtgtggatgatgtgggttccttggataacttagaggcattgtttgagcagagtacccagggatcattcattgacaatgatgaggtggatcagggaaattccttggcattgctttcagagcagttatttgcaactgatgagcaacagattgcagccctaaaacgaaagtatgccgcgacacctaagaaaaaaacggtagaaatcgaaaatctgagtcctagattagagtccgtcagcatttcacctaaaggaaagagcaggagacggttgtttgacagcggaataggacatgaaactcaagatactccttcggggagcgaggtacctatgagcatatctgggtctagttcagccaattcaagcataggaagccagtgcgagagcgagcaggtaaatagtaacactttgatttcttctgaagatttgcttagaacaagtaatagattggcagggtgctatgcgaggtttaaggaggcatttgggtgcagcttcaccgatctaacgcgtagctttaagagtgataagacatgtagtccgaattgggtcgtagctgtgtttggggctagagaacatttgttgcaggccttacatgatgtgtggaagaacacctatgagtactgccaagatacaacaagttatgcagggaatagaaaggtgaacttgctgcttatggagctgaaggtaggtaggagcagactcacattgcggagacagctttccgccatgttaggtgtggatgagttgttaatactcgccgatccgccgaacgagcggagcacgctcgccgcactttatttttataataaggttttatttaaaagtccttctaccatgttttacggtagcaccccgctgtggatagccagcaagacactactagagcatgctagtgcaacagccgagtcctttgatttcagtagtatggtgcagtgggcatatgacaatagactaaatgaggaggcagaaatagcttataaatatgccttagaagcagacagcaataagaatgcccaagcgtggcttaagactacaaaccaggtaaagcatgtccgagactgctgtgcaatggtcaggctatataacaggcaggaaatgaaggaaatgacaatggctcagtggatacggaagtgctgcgatgagacagaggaagaaggggactggaaggttattgcaaacttccttagataccaggaagtcaacctcatactgctgcttacagcacttaggcatatgtttaagggtactcctaaaaaacactgcctcgttatcacaggtcccccagatactgggaagtcatatttctgtaatagtctgaatgggtttcttaaaggtcgtgtaatttcatttatgaacagtaggagtcagttctggctgcagcctttagcagatgcaaaaatggggttcctagatgatgctacaaccgcttgctggaactttatggatgtataCatgcggaatgcattagatggcaatcccatgcagcttgacattaagcatagagcacctttgcagcttaagctacctccgctactaattacctcaaatgtagatgtcatgaataatgacaatttcagatatctacatagcaggttgcaggcctttgagtttcataagcctatgcctttaacagctaatgggcagccagtatatccccttactaaagctaattggaaatctttttttacaaggctggctaatcaattaggaatcgaagaggaggagggcgagaatgaacagcctggaaacacgtttcgatgcagtgcaagaccagatactgaacctttacgagaaaggcagtaaatgtttagcggaccacatactatattgggagcttgttaggaaagaaggagcattgcaattctgtgctcgtagagggggactcaacaagctcggactgcaacccctacccagcaccataggagctgagaacaaggccaaaagggcaattcagatgcaattggtgctaacatctctcaatgaatcaccctttggctccgaggagtggacaatggctgaaactagccgtgagatgtatgacagcactgagccgtatgggacttttaaaaaaagtggcgaggaggtggaagtctattatggaggagatgaagataataatgtgtcttatatgctctggaagtatgtctatgcccaggatgagaacggcaactggcataagtatcagagcgattgtgactattatggtgtacattacactgaccacagtgggacccgtatctattatcatgattttgacagtgattctcgcagatatggggattattctcactggactgtgaattataaacacaaaacttttgaatcttctcctgatagctcctcctcagccaaagaagggcatcaaaaaacaaccagacggcccgaagacaacaccgccacgaagagaactcttcccaccgacaccactgacacagccgcccccgccggagacaccatttggggacgaggcggaggagtacgactcggacaaggagaacgacaaacctgcatccggaaagcttggtcaagcgctgcagagactccagcaggacctgagggatctgcaggaccttgtcaaccaaacaacagccggcatcaccatactcataggccaataatctctgtcaaaggtccgactaactctttaaaatgctggcggaataggttgcgtcggagaacatataagccatatagccgtgtatctactgcctttcagtgggttgaggacagggcggacggggtagaggtgggggataggtggcaggttagctttagcaatgtacttgtagcttttgcagacacgtatcaaaaagaagtgtttctaaagactgtgacactgcccaagggctgctcatacaccagtggcttcttagacggactctgatagtggattctatacaccatccagaattactgtacctgttagattatttttgtaccattatggtgtctgctgacagaagcaggcgcgtcaagagggactctgcgtcaaacctatacagacaatgtcaagtaaccgggaattgtccacctgatgtagtcaataaagtcgaaggaaacacacttgctgacaggattcttaaagttattagtagcattgtatacttgggggggctgggcattggaactggcagaggctccgggggcaccactggctatgggcccataaactctgctggtggaagggtaacaggcacaggcacggtcatgaggcctggtgtcactgttgagcccattggcccaggggacatagtcactgtagactctgtgggccctggagattcgagccttattcctctacttgaggtgacccccgatgtccctataaatgggggacccgaggttccttctagtgggccagacataagcacagtggacgtgacatctagcatagacccaatatcagacctgtctgtgactggcaccacaatctccaacacagactctgctgtcattgatgttcagccatccccgggccctcgtagagtcataatcactagaagtgactttaataacccctcctatgtgtctgttgtgcaccccacacaggggttgggggagtctgggggtgtcattagtggagaaagtggaggcataatatccagcatacatgagctggataacaccacagtcataggtgctaggccaccacctgaaaggatattggatgaggtaccaggaccctttgaggacattgAgcttgacacatttgttgagtctagtggtcttagtgagtttgacatagagcagcccctcactagcacacctgaaggcccgttgcaaagggcggccactagattcagagacctgtataataggcgggtgcagcaggtgcgtgtatccaatccagaagcttttctaactggtcccagacaggcggtagtatttgaaaatcccgcctttgagcctgggagcctggattttgaacttcccgccagtcctcctgtagctgcacctgaccctgagtacactgatgtggtccacctagggcgtcagaggttctctgaggtgaacagagtaattagagtgagcaggttggggcaacgtgcatctatgaagactaggagtggtcttataattggtgggaaagtgcacttctatacagatttatcccctgttgctacggacattgaaatgcacacattaggtgagatcagtggtactgaagagctgattgatggtcttggaagctcttcagtaattgagttcccaaggggggttgagtctgtagagcttccagatggctctgactcagtgaatgagctacGtgacaccgatagtgctgatttttcttcctctaggcttgaactacttataggtaatgggacaagccgttttgtgatgcctgacttggtcgaaactctaggcccagacatgttttttcccagtatcgactcaggcacggttatacaccaccctcaagataattatgttcctattattctgccagctgcggatctattcccagcttctactgttataagtgtggatgatgactttgctgatttttatttgcaccccagtctccgtaaacgcaaacgaaaatatcgtatttattgatatttttcagatggcaatgtggacaccccagaccgggaagctttacctcccacctacaactccagtggcaaaagtgcagagcacagacgaatatgtgtaccctacgtctctcttctgtcatgcacacacggaccgtttgctaacagtgggccacccttttttttctgtcattgacaatgacaaggtcactgtgcctaaagtgtctggcaaccaatatagggttttcagacttaaattcccagatccaaataaatttgcattgccccaaaaggatttctatgatcctgagaaagaacggttagtgtggaggttaaggggtctggaaattggaagaggtggcccattagggattggcactaccgggcaccccctttttaacaagcttggagacacggaaaatccaaataaatatcagcaaggctctaaggataataggcagaacacttccatggaccccaaacaaacacagctgtttattgttggctgtgaaccccctacaggggaacactgggatgtagctaagccctgtggagctctggagaagggtgactgccctcctatccaacttgtaaatagtgtaattgaggatggggatatgtgtgacattggctttgggaatatgaacttcaaagagctgcagcaggataggagtggtgtgcctcttgatattgtatctacccggtgcaaatggcccgactttctgaaaatgaccaatgaggcatatggggataagatgttcttctttggaaggagagagcaagtgtatgcaagacactttttcaccaggaatggctctgtgggggagcccataccaaactctgtgagtcccagtgacttttactacgcacccgacagcacacaggaccagaagacactcgcaccctccgtgtactttggaactcctagtgggtcacttgtgtcgagtgatggtcagctgtttaacaggccattttggcttcaaagggctcagggaaacaataatggtgtgtgctggcacaatgagctctttgttactgttgtcgacaacacaaggaatacaaactttactatctcccagcaaaccaacacaccaaacccagatacatatgactctactaattttaaaaactatttaagacatgtggaacaatttgagctgtcccttattgctcaactgtgtaaggttccacttgacccgggtgtgcttgcccatataaacactatgaacccaaccatcttggagaactggaacttgggttttgtacctcccccacagcagtccatctctgatgactataggtatataacatcatcggcaactcgctgtccagatcagaatccgcccaaggaaagagaggatccttacaagggtcttatattttgggaagttgatcttactgagaggttttctcaggaccttgatcagtttgctctgggacgaaagtttctgtatcaagctggtatacgtactgctgttacgggccgcggggtcaaaagggcagcgtctacaacctctgcgtct***TCTAGA***

lox-cre-lox XhoI and XbaI digested

***TCTAGA***CGAGTTGTAAAACGGAAGAGGGGAAGCAAATAACTGAACTGGTGCTACTAACTGAATGACTCCGGTATTATGAAGTTCTTGTATTGTATAACTGTTTACTGGGGGCTTACTGTGTATAGGGGGCTTGAGTTGTTTGTCTGTTCTTGTCCATGTCCTTGTGATGTACTTTTGCAACTTAAATAAATGACTAATGCTGAATAACTTCGTATAGCATACATTATACGAAGTTATGAATTCGGGAACGGTATATGAACGGTGtcgacattgattattgactagttattaatagtaatcaattacggggtcattagttcatagcccatatatggagttccgcgttacataacttacggtaaatggcccgcctggctgaccgcccaacgacccccgcccattgacgtcaataatgacgtatgttcccatagtaacgccaatagggactttccattgacgtcaatgggtggagtatttacggtaaactgcccacttggcagtacatcaagtgtatcatatgccaagtacgccccctattgacgtcaatgacggtaaatggcccgcctggcattatgcccagtacatgaccttatgggactttcctacttggcagtacatctacgtattagtcatcgctattaccatggtcgaggtgagccccacgttctgcttcactctccccatctcccccccctccccacccccaattttgtatttatttattttttaattattttgtgcagcgatgggggcggggggggggggggcgcgcgccaggcggggggggggggggggggggggggggggggggggggcgggggggggcggcggcagccaatcagagcggcgcgctccgaaagtttccttttatggcgaggcggcggcggcggcggccctataaaaagcgaagcgcgcggcgggcgggagtcgctgcgcgctgccttcgccccgtgccccgctccgccgccgcctcgcgccgcccgccccggctctgactgaccgcgttactcccacaggtgagcgggcgggacggcccttctcctccgggctgtaattagcgcttggtttaatgacggcttgtttcttttctgtggctgcgtgaaagccttgaggggctccgggagggccctttgtgcggggggagcggctcggggggtgcgtgcgtgtgtgtgtgcgtggggagcgccgcgtgcggctccgcgctgcccggcggctgtgagcgctgcgggcgcggcgcggggctttgtgcgctccgcagtgtgcgcgaggggagcgcggccgggggcggtgccccgcggtgcggggggggctgcgaggggaacaaaggctgcgtgcggggtgtgtgcgtgggggggtgagcagggggtgtgggcgcgtcggtcgggctgcaaccccccctgcacccccctccccgagttgctgagcacggcccggcttcgggtgcggggctccgtacggggcgtggcgcggggctcgccgtgccgggcggggggtggcggcaggtgggggtgccgggcggggcggggccgcctcgggccggggagggctcgggggaggggcgcggcggcccccggagcgccggcggctgtcgaggcgcggcgagccgcagccattgccttttatggtaatcgtgcgagagggcgcagggacttcctttgtcccaaatctgtgcggagccgaaatctgggaggcgccgccgcaccccctctagcgggcgcggggcgaagcggtgcggcgccggcaggaaggaaatgggcggggagggccttcgtgcgtcgccgcgccgccgtccccttctccctctccagcctcggggctgtccgcggggggacggctgccttcgggggggacggggcagggcggggttcggcttctggcgtgtgaccggcggctctagctagagcctctgctaaccatgttcatgccttcttctttttcctacagctcctgggcaacgtgctggttattgtgctgtctcatcattttggcaaagaattctgagccgccaccatggccaatttactgaccgtacaccaaaatttgcctgcattaccggtcgatgcaacgagtgatgaggttcgcaagaacctgatggacatgttcagggatcgccaggcgttttctgagcatacctggaaaatgcttctgtccgtttgccggtcgtgggcggcatggtgcaagttgaataaccggaaatggtttcccgcagaacctgaagatgttcgcgattatcttctatatcttcaggcgcgcggtctggcagtaaaaactatccagcaacatttgggccagctaaacatgcttcatcgtcggtccgggctgccacgaccaagtgacagcaatgctgtttcactggttatgcggcggatccgaaaagaaaacgttgatgccggtgaacgtgcaaaacaggctctagcgttcgaacgcactgatttcgaccaggttcgttcactcatggaaaatagcgatcgctgccaggatatacgtaatctggcatttctggggattgcttataacaccctgttacgtatagccgaaattgccaggatcagggttaaagatatctcacgtactgacggtgggagaatgttaatccatattggcagaacgaaaacgctggttagcaccgcaggtgtagagaaggcacttagcctgggggtaactaaactggtcgagcgatggatttccgtctctggtgtagctgatgatccgaataactacctgttttgccgggtcagaaaaaatggtgttgccgcgccatctgccaccagccagctatcaactcgcgccctggaagggatttttgaagcaactcatcgattgatttacggcgctaaggatgactctggtcagagatacctggcctggtctggacacagtgcccgtgtcggagccgcgcgagatatggcccgcgctggagtttcaataccggagatcatgcaagctggtggctggaccaatgtaaatattgtcatgaactatatccgtaacctggatagtgaaacaggggcaatggtgcgcctgctggaagatggcgatggaccggtggaacaaaaacttatttctgaagaagatctgtgatagcggccgcactcctcaggtgcaggctgcctatcagaaggtggtggctggtgtggccaatgccctggctcacaaataccactgagatctttttccctctgccaaaaattatggggacatcatgaagccccttgagcatctgacttctggctaataaaggaaatttattttcattgcaatagtgtgttggaattttttgtgtctctcactcggaaggacatatgggagggcaaatcatttaaaacatcagaatgagtatttggtttagagtttggcaacatatgcccatatgctggctgccatgaacaaaggttggctataaagaggtcatcagtatatgaaacagccccctgctgtccattccttattccatagaaaagccttgacttgaggttagattttttttatattttgttttgtgttatttttttctttaacatccctaaaattttccttacatgttttactagccagatttttcctcctctcctgactactcccagtcatagctgtccctcttctcttatggagatccctcgacctgcagCCAGTGAGCGCGACGTAATACGACTCACTATAGGGCGAATTGGCGGAAGGCCGTCAAGGCCACGTGTCTTGTCCAGAGCTCGGGAACGGTGAATTCATAACTTCGTATAGCATACATTATACGAAGTTACCAGTGTGCCTCGCCTCATTCTTTAGCTCGCACCTGGGCTCACTTTGTGCCAGACTGTCATAACAAACAGTCTCTGTTGGCTGTGTGCTCTCTAATTTCTCGAAAAGACGTGTTTTGACGAAGGACCGTTTTCGGTCGGGCGCCAGTATCAGCATAAACTCCAGCCAATTTGGCCAAGGTAAGGAAATGACTAACTGTCTTGGAACAGATGCGTGTCCTGGCAATTATCCGCGTACCGTTTTCGGTCGGGTAAAAAAGGCGCCAAGCTAAGCATGATTCAGAGTTCCATTGTGTTCTGCCAAGTACAGGTGTGGTGTTCTGGAACGGTCGTACAATTAATCTTTGAGCTGATGGTTGGCAACAATTATTTCCCTCTGAAAAAATTTAGGTGGAGCGGGAACGGTCGCATATAAGTATCAGTGTGCCCCCATAACCGTATTCGTTCATGGAAATCGGCAAAGGCTACACT***CTCGAG***
