# Supplementary material for: A novel lineage-tracing mouse model for studying early MmuPV1 infections
Source: eLife. 2022 May 9;11:e72638. doi: 10.7554/eLife.72638 (PMC9084889; doi:10.7554/eLife.72638)
Supplement: Source data 2. [file elife-72638-data2.zip › Sequences and Plasmid creation/MmuPV1-lox-Cre-lox Final Sequence.docx]

MmuPV1-lox-Cre-lox Sequence

CGAGTTGTAAAACGGAAGAGGGGAAGCAAATAACTGAACTGGTGCTACTAACTGAATGACTCCGGTATTATGAAGTTCTTGTATTGTATAACTGTTTACTGGGGGCTTACTGTGTATAGGGGGCTTGAGTTGTTTGTCTGTTCTTGTCCATGTCCTTGTGATGTACTTTTGCAACTTAAATAAATGACTAATGCTGAATAACTTCGTATAGCATACATTATACGAAGTTATGAATTCGGGAACGGTATATGAACGGTGgtactcgacattgattattgactagttattaatagtaatcaattacggggtcattagttcatagcccatatatggagttccgcgttacataacttacggtaaatggcccgcctggctgaccgcccaacgacccccgcccattgacgtcaataatgacgtatgttcccatagtaacgccaatagggactttccattgacgtcaatgggtggagtatttacggtaaactgcccacttggcagtacatcaagtgtatcatatgccaagtacgccccctattgacgtcaatgacggtaaatggcccgcctggcattatgcccagtacatgaccttatgggactttcctacttggcagtacatctacgtattagtcatcgctattaccatggtcgaggtgagccccacgttctgcttcactctccccatctcccccccctccccacccccaattttgtatttatttattttttaattattttgtgcagcgatgggggcggggggggggggggcgcgcgccaggcggggggggggggggggggggggggggggggggggggcgggggggggcggcggcagccaatcagagcggcgcgctccgaaagtttccttttatggcgaggcggcggcggcggcggccctataaaaagcgaagcgcgcggcgggcgggagtcgctgcgcgctgccttcgccccgtgccccgctccgccgccgcctcgcgccgcccgccccggctctgactgaccgcgttactcccacaggtgagcgggcgggacggcccttctcctccgggctgtaattagcgcttggtttaatgacggcttgtttcttttctgtggctgcgtgaaagccttgaggggctccgggagggccctttgtgcggggggagcggctcggggggtgcgtgcgtgtgtgtgtgcgtggggagcgccgcgtgcggctccgcgctgcccggcggctgtgagcgctgcgggcgcggcgcggggctttgtgcgctccgcagtgtgcgcgaggggagcgcggccgggggcggtgccccgcggtgcggggggggctgcgaggggaacaaaggctgcgtgcggggtgtgtgcgtgggggggtgagcagggggtgtgggcgcgtcggtcgggctgcaaccccccctgcacccccctccccgagttgctgagcacggcccggcttcgggtgcggggctccgtacggggcgtggcgcggggctcgccgtgccgggcggggggtggcggcaggtgggggtgccgggcggggcggggccgcctcgggccggggagggctcgggggaggggcgcggcggcccccggagcgccggcggctgtcgaggcgcggcgagccgcagccattgccttttatggtaatcgtgcgagagggcgcagggacttcctttgtcccaaatctgtgcggagccgaaatctgggaggcgccgccgcaccccctctagcgggcgcggggcgaagcggtgcggcgccggcaggaaggaaatgggcggggagggccttcgtgcgtcgccgcgccgccgtccccttctccctctccagcctcggggctgtccgcggggggacggctgccttcgggggggacggggcagggcggggttcggcttctggcgtgtgaccggcggctCTAGctagagcctctgctaaccatgttcatgccttcttctttttcctacagctcctgggcaacgtgctggttattgtgctgtctcatcattttggcaaagaattctgagccgccaccatggccaatttactgaccgtacaccaaaatttgcctgcattaccggtcgatgcaacgagtgatgaggttcgcaagaacctgatggacatgttcagggatcgccaggcgttttctgagcatacctggaaaatgcttctgtccgtttgccggtcgtgggcggcatggtgcaagttgaataaccggaaatggtttcccgcagaacctgaagatgttcgcgattatcttctatatcttcaggcgcgcggtctggcagtaaaaactatccagcaacatttgggccagctaaacatgcttcatcgtcggtccgggctgccacgaccaagtgacagcaatgctgtttcactggttatgcggcggatccgaaaagaaaacgttgatgccggtgaacgtgcaaaacaggctctagcgttcgaacgcactgatttcgaccaggttcgttcactcatggaaaatagcgatcgctgccaggatatacgtaatctggcatttctggggattgcttataacaccctgttacgtatagccgaaattgccaggatcagggttaaagatatctcacgtactgacggtgggagaatgttaatccatattggcagaacgaaaacgctggttagcaccgcaggtgtagagaaggcacttagcctgggggtaactaaactggtcgagcgatggatttccgtctctggtgtagctgatgatccgaataactacctgttttgccgggtcagaaaaaatggtgttgccgcgccatctgccaccagccagctatcaactcgcgccctggaagggatttttgaagcaactcatcgattgatttacggcgctaaggatgactctggtcagagatacctggcctggtctggacacagtgcccgtgtcggagccgcgcgagatatggcccgcgctggagtttcaataccggagatcatgcaagctggtggctggaccaatgtaaatattgtcatgaactatatccgtaacctggatagtgaaacaggggcaatggtgcgcctgctggaagatggcgatggaccggtggaacaaaaacttatttctgaagaagatctgtgatagcggccgcactcctcaggtgcaggctgcctatcagaaggtggtggctggtgtggccaatgccctggctcacaaataccactgagatctttttccctctgccaaaaattatggggacatcatgaagccccttgagcatctgacttctggctaataaaggaaatttattttcattgcaatagtgtgttggaattttttgtgtctctcactcggaaggacatatgggagggcaaatcatttaaaacatcagaatgagtatttggtttagagtttggcaacatatgcccatatgctggctgccatgaacaaaggttggctataaagaggtcatcagtatatgaaacagccccctgctgtccattccttattccatagaaaagccttgacttgaggttagattttttttatattttgttttgtgttatttttttctttaacatccctaaaattttccttacatgttttactagccagatttttcctcctctcctgactactcccagtcatagctgtccctcttctcttatggagatccctcgacctgcagCCAGTGAGCGCGACGTAATACGACTCACTATAGGGCGAATTGGCGGAAGGCCGTCAAGGCCACGTGTCTTGTCCAGAGCTCGGGAACGGTGAATTCATAACTTCGTATAGCATACATTATACGAAGTTACCAGTGTGCCTCGCCTCATTCTTTAGCTCGCACCTGGGCTCACTTTGTGCCAGACTGTCATAACAAACAGTCTCTGTTGGCTGTGTGCTCTCTAATTTCTCGAAAAGACGTGTTTTGACGAAGGACCGTTTTCGGTCGGGCGCCAGTATCAGCATAAACTCCAGCCAATTTGGCCAAGGTAAGGAAATGACTAACTGTCTTGGAACAGATGCGTGTCCTGGCAATTATCCGCGTACCGTTTTCGGTCGGGTAAAAAAGGCGCCAAGCTAAGCATGATTCAGAGTTCCATTGTGTTCTGCCAAGTACAGGTGTGGTGTTCTGGAACGGTCGTACAATTAATCTTTGAGCTGATGGTTGGCAACAATTATTTCCCTCTGAAAAAATTTAGGTGGAGCGGGAACGGTCGCATATAAGTATCAGTGTGCCCCCATAACCGTATTCGTTCATGGAAATCGGCAAAGGCTACACTCTCGAGgaggtgcttagatattctaacaaagatgtcgtggattttcatttgtcttgtgctttttgctctactactatggatcataacgagaaggccagattcatacaggctaaattgaaatgtgttgttagagattttgcttttaaaggtgcttgtattgtgtgccgcagacagcttgcttgcaaggaaaagcttttgcatactagagttacaggggaggctgatttggtagagtgcatggctggcaagaatattgtgtttgttactgtaagatgtgttacgtgcctggcactccttactgcctctgaaaagcttgatgccaaagcgtgcggcttgccatttcacttggtgcgccacatgtggagaggctactgcgggttctgcaaaccattactataatgcagggcccattaccaacaattgctgacatcgagattcagaatctcgactcacttttgggtgttggtgagcctgacctacccgatgttgggtcatcatcgttgtcaccagactcgttaggagaagaggaggagctggagctggagactatcgatgtagatccttacaggattaaaacaacctgcttttgctgcgacactgttctccggttcataattgtgaccggagacgactcggtgaaagcattcgagtcactgcttctgcaggatcttagctttgtctgcccgcactgcgtcgcgtcgtacgtgaacctcagaaatggaaaacgataaaggtacagggcagtattctggatggtgttttatagataatgaggctgaatgtgtggatgatgtgggttccttggataacttagaggcattgtttgagcagagtacccagggatcattcattgacaatgatgaggtggatcagggaaattccttggcattgctttcagagcagttatttgcaactgatgagcaacagattgcagccctaaaacgaaagtatgccgcgacacctaagaaaaaaacggtagaaatcgaaaatctgagtcctagattagagtccgtcagcatttcacctaaaggaaagagcaggagacggttgtttgacagcggaataggacatgaaactcaagatactccttcggggagcgaggtacctatgagcatatctgggtctagttcagccaattcaagcataggaagccagtgcgagagcgagcaggtaaatagtaacactttgatttcttctgaagatttgcttagaacaagtaatagattggcagggtgctatgcgaggtttaaggaggcatttgggtgcagcttcaccgatctaacgcgtagctttaagagtgataagacatgtagtccgaattgggtcgtagctgtgtttggggctagagaacatttgttgcaggccttacatgatgtgtggaagaacacctatgagtactgccaagatacaacaagttatgcagggaatagaaaggtgaacttgctgcttatggagctgaaggtaggtaggagcagactcacattgcggagacagctttccgccatgttaggtgtggatgagttgttaatactcgccgatccgccgaacgagcggagcacgctcgccgcactttatttttataataaggttttatttaaaagtccttctaccatgttttacggtagcaccccgctgtggatagccagcaagacactactagagcatgctagtgcaacagccgagtcctttgatttcagtagtatggtgcagtgggcatatgacaatagactaaatgaggaggcagaaatagcttataaatatgccttagaagcagacagcaataagaatgcccaagcgtggcttaagactacaaaccaggtaaagcatgtccgagactgctgtgcaatggtcaggctatataacaggcaggaaatgaaggaaatgacaatggctcagtggatacggaagtgctgcgatgagacagaggaagaaggggactggaaggttattgcaaacttccttagataccaggaagtcaacctcatactgctgcttacagcacttaggcatatgtttaagggtactcctaaaaaacactgcctcgttatcacaggtcccccagatactgggaagtcatatttctgtaatagtctgaatgggtttcttaaaggtcgtgtaatttcatttatgaacagtaggagtcagttctggctgcagcctttagcagatgcaaaaatggggttcctagatgatgctacaaccgcttgctggaactttatggatgtataCatgcggaatgcattagatggcaatcccatgcagcttgacattaagcatagagcacctttgcagcttaagctacctccgctactaattacctcaaatgtagatgtcatgaataatgacaatttcagatatctacatagcaggttgcaggcctttgagtttcataagcctatgcctttaacagctaatgggcagccagtatatccccttactaaagctaattggaaatctttttttacaaggctggctaatcaattaggaatcgaagaggaggagggcgagaatgaacagcctggaaacacgtttcgatgcagtgcaagaccagatactgaacctttacgagaaaggcagtaaatgtttagcggaccacatactatattgggagcttgttaggaaagaaggagcattgcaattctgtgctcgtagagggggactcaacaagctcggactgcaacccctacccagcaccataggagctgagaacaaggccaaaagggcaattcagatgcaattggtgctaacatctctcaatgaatcaccctttggctccgaggagtggacaatggctgaaactagccgtgagatgtatgacagcactgagccgtatgggacttttaaaaaaagtggcgaggaggtggaagtctattatggaggagatgaagataataatgtgtcttatatgctctggaagtatgtctatgcccaggatgagaacggcaactggcataagtatcagagcgattgtgactattatggtgtacattacactgaccacagtgggacccgtatctattatcatgattttgacagtgattctcgcagatatggggattattctcactggactgtgaattataaacacaaaacttttgaatcttctcctgatagctcctcctcagccaaagaagggcatcaaaaaacaaccagacggcccgaagacaacaccgccacgaagagaactcttcccaccgacaccactgacacagccgcccccgccggagacaccatttggggacgaggcggaggagtacgactcggacaaggagaacgacaaacctgcatccggaaagcttggtcaagcgctgcagagactccagcaggacctgagggatctgcaggaccttgtcaaccaaacaacagccggcatcaccatactcataggccaataatctctgtcaaaggtccgactaactctttaaaatgctggcggaataggttgcgtcggagaacatataagccatatagccgtgtatctactgcctttcagtgggttgaggacagggcggacggggtagaggtgggggataggtggcaggttagctttagcaatgtacttgtagcttttgcagacacgtatcaaaaagaagtgtttctaaagactgtgacactgcccaagggctgctcatacaccagtggcttcttagacggactctgatagtggattctatacaccatccagaattactgtacctgttagattatttttgtaccattatggtgtctgctgacagaagcaggcgcgtcaagagggactctgcgtcaaacctatacagacaatgtcaagtaaccgggaattgtccacctgatgtagtcaataaagtcgaaggaaacacacttgctgacaggattcttaaagttattagtagcattgtatacttgggggggctgggcattggaactggcagaggctccgggggcaccactggctatgggcccataaactctgctggtggaagggtaacaggcacaggcacggtcatgaggcctggtgtcactgttgagcccattggcccaggggacatagtcactgtagactctgtgggccctggagattcgagccttattcctctacttgaggtgacccccgatgtccctataaatgggggacccgaggttccttctagtgggccagacataagcacagtggacgtgacatctagcatagacccaatatcagacctgtctgtgactggcaccacaatctccaacacagactctgctgtcattgatgttcagccatccccgggccctcgtagagtcataatcactagaagtgactttaataacccctcctatgtgtctgttgtgcaccccacacaggggttgggggagtctgggggtgtcattagtggagaaagtggaggcataatatccagcatacatgagctggataacaccacagtcataggtgctaggccaccacctgaaaggatattggatgaggtaccaggaccctttgaggacattgAgcttgacacatttgttgagtctagtggtcttagtgagtttgacatagagcagcccctcactagcacacctgaaggcccgttgcaaagggcggccactagattcagagacctgtataataggcgggtgcagcaggtgcgtgtatccaatccagaagcttttctaactggtcccagacaggcggtagtatttgaaaatcccgcctttgagcctgggagcctggattttgaacttcccgccagtcctcctgtagctgcacctgaccctgagtacactgatgtggtccacctagggcgtcagaggttctctgaggtgaacagagtaattagagtgagcaggttggggcaacgtgcatctatgaagactaggagtggtcttataattggtgggaaagtgcacttctatacagatttatcccctgttgctacggacattgaaatgcacacattaggtgagatcagtggtactgaagagctgattgatggtcttggaagctcttcagtaattgagttcccaaggggggttgagtctgtagagcttccagatggctctgactcagtgaatgagctacGtgacaccgatagtgctgatttttcttcctctaggcttgaactacttataggtaatgggacaagccgttttgtgatgcctgacttggtcgaaactctaggcccagacatgttttttcccagtatcgactcaggcacggttatacaccaccctcaagataattatgttcctattattctgccagctgcggatctattcccagcttctactgttataagtgtggatgatgactttgctgatttttatttgcaccccagtctccgtaaacgcaaacgaaaatatcgtatttattgatatttttcagatggcaatgtggacaccccagaccgggaagctttacctcccacctacaactccagtggcaaaagtgcagagcacagacgaatatgtgtaccctacgtctctcttctgtcatgcacacacggaccgtttgctaacagtgggccacccttttttttctgtcattgacaatgacaaggtcactgtgcctaaagtgtctggcaaccaatatagggttttcagacttaaattcccagatccaaataaatttgcattgccccaaaaggatttctatgatcctgagaaagaacggttagtgtggaggttaaggggtctggaaattggaagaggtggcccattagggattggcactaccgggcaccccctttttaacaagcttggagacacggaaaatccaaataaatatcagcaaggctctaaggataataggcagaacacttccatggaccccaaacaaacacagctgtttattgttggctgtgaaccccctacaggggaacactgggatgtagctaagccctgtggagctctggagaagggtgactgccctcctatccaacttgtaaatagtgtaattgaggatggggatatgtgtgacattggctttgggaatatgaacttcaaagagctgcagcaggataggagtggtgtgcctcttgatattgtatctacccggtgcaaatggcccgactttctgaaaatgaccaatgaggcatatggggataagatgttcttctttggaaggagagagcaagtgtatgcaagacactttttcaccaggaatggctctgtgggggagcccataccaaactctgtgagtcccagtgacttttactacgcacccgacagcacacaggaccagaagacactcgcaccctccgtgtactttggaactcctagtgggtcacttgtgtcgagtgatggtcagctgtttaacaggccattttggcttcaaagggctcagggaaacaataatggtgtgtgctggcacaatgagctctttgttactgttgtcgacaacacaaggaatacaaactttactatctcccagcaaaccaacacaccaaacccagatacatatgactctactaattttaaaaactatttaagacatgtggaacaatttgagctgtcccttattgctcaactgtgtaaggttccacttgacccgggtgtgcttgcccatataaacactatgaacccaaccatcttggagaactggaacttgggttttgtacctcccccacagcagtccatctctgatgactataggtatataacatcatcggcaactcgctgtccagatcagaatccgcccaaggaaagagaggatccttacaagggtcttatattttgggaagttgatcttactgagaggttttctcaggaccttgatcagtttgctctgggacgaaagtttctgtatcaagctggtatacgtactgctgttacgggccgcggggtcaaaagggcagcgtctacaacctctgcgtcttCTAGA
