# Supplementary material for: A novel lineage-tracing mouse model for studying early MmuPV1 infections
Source: eLife. 2022 May 9;11:e72638. doi: 10.7554/eLife.72638 (PMC9084889; doi:10.7554/eLife.72638)
Supplement: Transparent reporting form [file elife-72638-transrepform1.pdf]

eLIFE
1st Floor
24 Hills Road
Cambridge CB2 1JP, UK
P 01223 855340
W elifesciences.org
T @elife

eLife's transparent reporting form

We encourage authors to provide detailed information *within their submission* to facilitate the interpretation and replication of experiments. Authors can upload supporting documentation to indicate the use of appropriate reporting guidelines for health-related research (see EQUATOR Network), life science research (see the BioSharing Information Resource), or the ARRIVE guidelines for reporting work involving animal research. Where applicable, authors should refer to any relevant reporting standards documents in this form.

If you have any questions, please consult our Journal Policies and/or contact us: editorial@elifesciences.org.

Sample-size estimation

- You should state whether an appropriate sample size was computed when the study was being designed
- You should state the statistical method of sample size computation and any required assumptions
- If no explicit power analysis was used, you should describe how you decided what sample (replicate) size (number) to use

Please outline where this information can be found within the submission (e.g., sections or figure legends), or explain why this information doesn't apply to your submission:

Since the primary goal of this paper is to generate and validate a tool/resource, no specific power analysis was used to estimate sample size. Instead, for validation of tools generated experiments were repeated two times (Figure 1) and representative results are shown. For studies describing longitudinal monitoring of mouse tissues for YFP, papillomavirus transcript expression, or flow cytometry analysis, 3 mice per group per time point were used consistent with similar recent studies (Sexual transmission of murine papillomavirus (MmuPV1) in *Mus musculus. Spurgeon and Lambert Elife 2019*).

Replicates

- You should report how often each experiment was performed
- You should include a definition of biological versus technical replication
- The data obtained should be provided and sufficient information should be provided to indicate the number of independent biological and/or technical replicates
- If you encountered any outliers, you should describe how these were handled
- Criteria for exclusion/inclusion of data should be clearly stated
- High-throughput sequence data should be uploaded before submission, with a private link for reviewers provided (these are available from both GEO and ArrayExpress)

Please outline where this information can be found within the submission (e.g., sections or figure legends), or explain why this information doesn't apply to your submission:

In Figure 1 experiments showing expression in cells were performed at least 2 times. In the rest of the figures each time point/condition includes 3 mice, and experiments were repeated at least twice. We define a biological replicate as a biologically distinct sample e.g., a mouse. For Figure 4 which contains quantitative information derived from flow cytometry all analyzed data were included, and none were handled as outliers.

Statistical reporting

- Statistical analysis methods should be described and justified
- Raw data should be presented in figures whenever informative to do so (typically when N per group is less than 10)
- For each experiment, you should identify the statistical tests used, exact values of N, definitions of center, methods of multiple test correction, and dispersion and precision measures (e.g., mean, median, SD, SEM, confidence intervals; and, for the major substantive results, a measure of effect size (e.g., Pearson's r, Cohen's d)
- Report exact p-values wherever possible alongside the summary statistics and 95% confidence intervals. These should be reported for all key questions and not only when the p-value is less than 0.05.

Please outline where this information can be found within the submission (e.g., sections or figure legends), or explain why this information doesn't apply to your submission:

Methods used for statistical analysis are included in the Materials and Methods section. Results of statistical analysis are reported in the relevant figure legends.

(For large datasets, or papers with a very large number of statistical tests, you may upload a single table file with tests, Ns, etc., with reference to sections in the manuscript.)

Group allocation

- Indicate how samples were allocated into experimental groups (in the case of clinical studies, please specify allocation to treatment method); if randomization was used, please also state if restricted randomization was applied
- Indicate if masking was used during group allocation, data collection and/or data analysis

Please outline where this information can be found within the submission (e.g., sections or figure legends), or explain why this information doesn't apply to your submission:

Mice were randomized into specific test/time point condition. Masking of slides was used during examination of samples in microscopy.

Additional data files ("source data")

- We encourage you to upload relevant additional data files, such as numerical data that are represented as a graph in a figure, or as a summary table
- Where provided, these should be in the most useful format, and they can be uploaded as "Source data" files linked to a main figure or table
- Include model definition files including the full list of parameters used
- Include code used for data analysis (e.g., R, MatLab)
- Avoid stating that data files are "available upon request"

Please indicate the figures or tables for which source data files have been provided:

eLife Sciences Publications, Ltd is a limited liability non-profit non-stock corporation incorporated in the State of Delaware, USA, with company number 5030732, and is registered in the UK with company number FC030576 and branch number BR015634 at the address 1st Floor, 24 Hills Road, Cambridge CB2 1JP | August 2014

Source data for gels in Figure 1, 3 and Figure 3 supplement 1 are provided in a ZIP file called "Source data Gels". Source data for sequences and plasmid creation in detail are provided in a ZIP file called "Source data Sequences and Plasmid creation". The source data of FACS for Figure 4 and its supplements is included in a Microsoft Excel spreadsheet called "Source data - FACS ". Additionally, figure supplements Fig 3 Supplement 1, Figure 4 Supplement 1 and Figure 4 Supplement 2 with their figure legends are provided in a word document called "Supp. Figures and Legends".

eLife Sciences Publications, Ltd is a limited liability non-profit non-stock corporation incorporated in the State of Delaware, USA, with company number 5030732, and is registered in the UK with company number FC030576 and branch number BR015634 at the address 1st Floor, 24 Hills Road, Cambridge CB2 1JP | August 2014
